# Tachycardiomyopathy entails a dysfunctional pattern of interrelated mitochondrial functions

**DOI:** 10.1007/s00395-022-00949-0

**Published:** 2022-09-06

**Authors:** Michael G. Paulus, Kathrin Renner, Alexander G. Nickel, Christoph Brochhausen, Katharina Limm, Elmar Zügner, Maria J. Baier, Steffen Pabel, Stefan Wallner, Christoph Birner, Andreas Luchner, Christoph Magnes, Peter J. Oefner, Klaus J. Stark, Stefan Wagner, Christoph Maack, Lars S. Maier, Katrin Streckfuss-Bömeke, Samuel Sossalla, Alexander Dietl

**Affiliations:** 1 Department of Internal Medicine II, University Hospital Regensburg, Regensburg, Germany; 2 Department of Internal Medicine III, University Hospital Regensburg, Regensburg, Germany; 3 Department of Translational Research, Comprehensive Heart Failure Center (CHFC), University Hospital Würzburg, Würzburg, Germany; 4 Institute of Pathology, University of Regensburg, Regensburg, Germany; 5 Institute of Functional Genomics, University of Regensburg, Regensburg, Germany; 6 Joanneum Research Health, Graz, Austria; 7 Institute of Clinical Chemistry and Laboratory Medicine, University Hospital Regensburg, Regensburg, Germany; 8 Department of Internal Medicine I, Klinikum St. Marien, Amberg, Germany; 9 Clinic for Cardiology, Krankenhaus der Barmherzigen Brüder, Regensburg, Germany; 10 Department of Genetic Epidemiology, University of Regensburg, Regensburg, Germany; 11 Clinic for Cardiology and Pneumology, Georg-August-University Göttingen, and DZHK (German Centre for Cardiovascular Research), Partner Site Göttingen, Göttingen, Germany; 12 Institute of Pharmacology and Toxicology, University of Würzburg, Würzburg, Germany

**Keywords:** Tachycardiomyopathy, Mitochondria, Redox, Acetylome

## Abstract

Tachycardiomyopathy is characterised by reversible left ventricular dysfunction, provoked by rapid ventricular rate. While the knowledge of mitochondria advanced in most cardiomyopathies, mitochondrial functions await elucidation in tachycardiomyopathy. Pacemakers were implanted in 61 rabbits. Tachypacing was performed with 330 bpm for 10 days (*n* = 11, early left ventricular dysfunction) or with up to 380 bpm over 30 days (*n* = 24, tachycardiomyopathy, TCM). In *n* = 26, pacemakers remained inactive (SHAM). Left ventricular tissue was subjected to respirometry, metabolomics and acetylomics. Results were assessed for translational relevance using a human-based model: induced pluripotent stem cell derived cardiomyocytes underwent field stimulation for 7 days (TACH–iPSC–CM). TCM animals showed systolic dysfunction compared to SHAM (fractional shortening 37.8 ± 1.0% vs. 21.9 ± 1.2%, SHAM vs. TCM, *p* < 0.0001). Histology revealed cardiomyocyte hypertrophy (cross-sectional area 393.2 ± 14.5 µm^2^ vs. 538.9 ± 23.8 µm^2^, *p* < 0.001) without fibrosis. Mitochondria were shifted to the intercalated discs and enlarged. Mitochondrial membrane potential remained stable in TCM. The metabolite profiles of ELVD and TCM were characterised by profound depletion of tricarboxylic acid cycle intermediates. Redox balance was shifted towards a more oxidised state (ratio of reduced to oxidised nicotinamide adenine dinucleotide 10.5 ± 2.1 vs. 4.0 ± 0.8, *p* < 0.01). The mitochondrial acetylome remained largely unchanged. Neither TCM nor TACH–iPSC–CM showed relevantly increased levels of reactive oxygen species. Oxidative phosphorylation capacity of TCM decreased modestly in skinned fibres (168.9 ± 11.2 vs. 124.6 ± 11.45 pmol·O_2_·s^−1^·mg^−1^ tissue, *p* < 0.05), but it did not in isolated mitochondria. The pattern of mitochondrial dysfunctions detected in two models of tachycardiomyopathy diverges from previously published characteristic signs of other heart failure aetiologies.

**Supplementary Information:**

The online version contains supplementary material available at 10.1007/s00395-022-00949-0.

## Introduction

Tachycardiomyopathy is characterised by reversible left ventricular (LV) dysfunction, induced by rapid ventricular rate [40]. Its prevalence is strikingly underappreciated, affecting about one third of patients with simultaneously diagnosed arrhythmia and systolic heart failure in treatment studies [39, 70]. While tachycardiomyopathy is often classified as dilated cardiomyopathy [75], it implies very specific remodelling processes, including a unique shift of mitochondria to the intercalated discs [59]. In heart failure research, the knowledge of mitochondria as a pathophysiological factor has considerably advanced in the past few years: the initial perception of mitochondrial signalling via reactive oxygen species (ROS) developed from a mere overwhelming harmful ROS production (oxidative stress) to a multi-faceted picture of a finely tuned ROS-answer in progressive heart failure, which is closely related to mitochondrial bioenergetics [19]. The balance between reduced and oxidised pyridine nucleotides (mitochondrial redox-balance) links ROS-emission to mitochondrial energetics [61, 63] and regulates the mitochondrial acetylome via NAD^+^-dependent deacetylases [45, 50, 71, 80]. By revealing these regulatory mechanisms, increasing layers of complexity have been added to the understanding of mitochondrial tasks. Previous studies in tachycardiomyopathy were restricted to the examination of narrow aspects of mitochondrial functions and did not take their complex interplay into consideration [42, 46, 56]. Thus, the interrelated multiplicity of mitochondrial functions in tachycardiomyopathy still awaits comprehensive elucidation.

To address this knowledge gap, we set out to analyse mitochondrial energetics, redox-balance, and signalling via ROS and post-translational acetylation by a descriptive systems medicine approach in an animal model of tachycardiomyopathy [11, 21]. Furthermore, we evaluated the translational relevance in a new model of long-term tachypaced human induced pluripotent stem cell derived cardiomyocytes (iPSC–CM). Together, we report the first integrative view on mitochondrial functions in tachycardiomyopathy.

## Methods

A detailed description of all methods is embedded in the Supplementary Information. All human and animal studies had been approved by the appropriate ethics committee and were performed in accordance with the ethical standards laid down in the 1964 Declaration of Helsinki and its later amendments.

### Animal model of tachycardiomyopathy

**Fig. 1:**
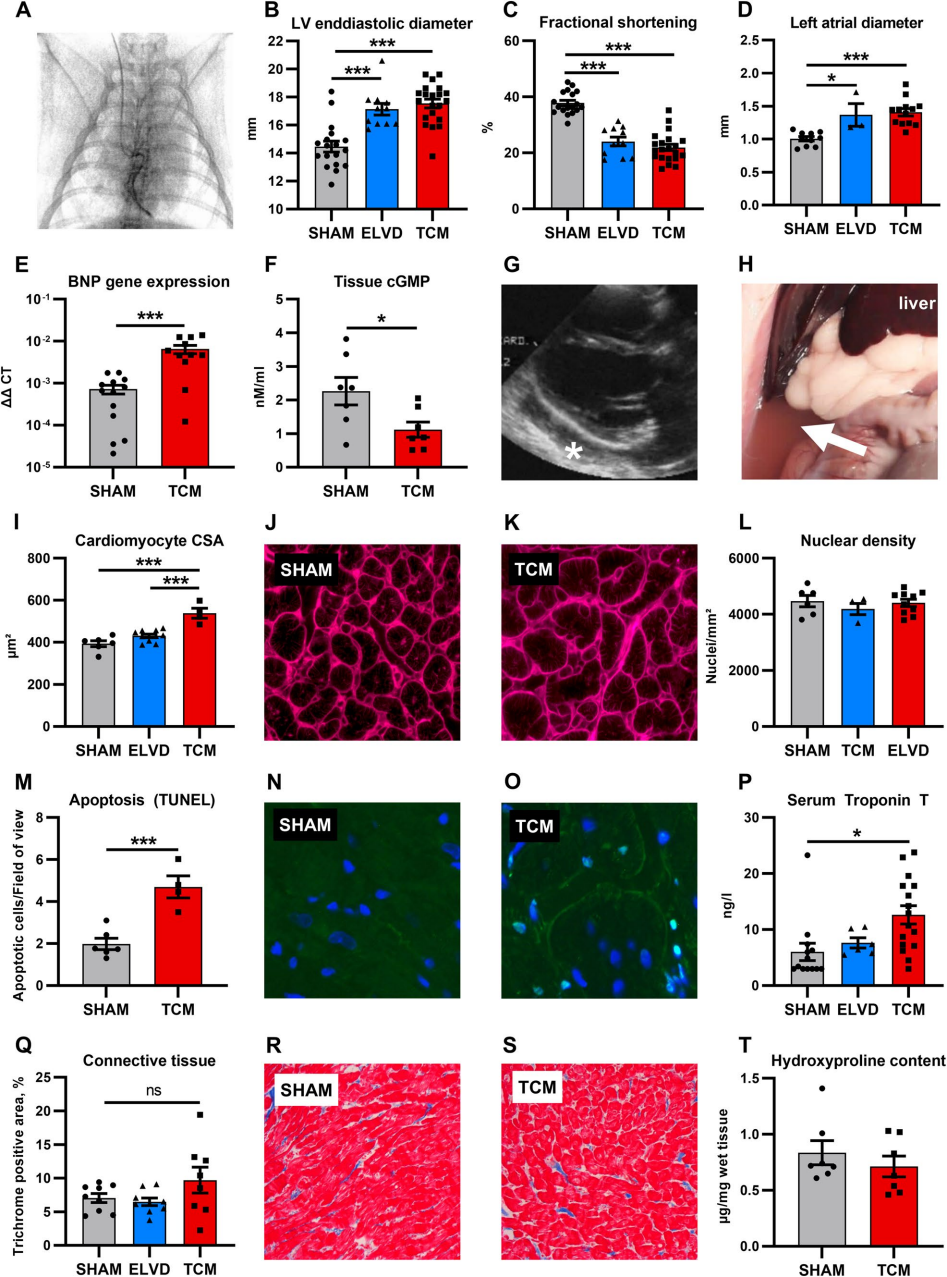
Tachypacing induces left ventricular non-fibrotic eccentric hypertrophy and severe heart failure syndrome. After 10 days of rapid ventricular pacing by a permanent pacemaker (**A**), animals developed progressive LV dilatation (**B**), systolic dysfunction (**C**) and left atrial enlargement (**D**). TCM animals showed severe systolic heart failure as evident in increased natriuretic peptide expression (**E**), reduced tissue cGMP levels (**F**), as well as fluid retention with pericardial effusion (**G**, asterisk) and ascites (**H**, arrow). Cardiomyocyte hypertrophy was confined to advanced disease with increased cell cross-sectional area and stable nuclear density (**I**–**L**). Apoptosis increased in TCM (**M**–**O**), resulting in increased high-sensitivity serum troponin T levels (**P**). Masson’s trichrome stain (**Q**–**S**) and measurement of hydroxyproline content (**T**) did not reveal any increase in fibrosis. Data are shown as mean ± SEM. *p < 0.05, ***p < 0.001 for ANOVA with Tukey post-hoc test (**B**–**D**, **I**, **P**) and *t* test (**F**, **M**). **A** fluoroscopy after placement of the right ventricular pacemaker lead. **J**, **K** wheat germ agglutinin staining. **N**, **O** staining with DAPI (blue) and TUNEL (green). **R**, **S** Masson’s trichrome staining. *cGMP* cyclic guanosine monophosphate, *CSA* cross-sectional area, *LV* left ventricular

For in vivo investigation, tachycardiomyopathy was induced in rabbits [21] (Supplementary Fig. 1). In brief, a total of 61 male New Zealand White rabbits underwent permanent pacemaker implantation (Fig. 1A, Advisa DR MRI SureScan, Medtronic, Minneapolis, MN, USA). After recovery, 24 rabbits underwent incremental tachypacing with up to 380 bpm for 30 days (tachycardiomyopathy, TCM). For investigation of the early disease stage, tachypacing with 330 bpm for 10 days was performed in 11 rabbits (early left ventricular dysfunction, ELVD). In 26 rabbits, the pacemaker remained inactive, serving as a control group (SHAM). Cardiac morphology and function were assessed by transthoracic echocardiography. The animal study was approved by the institutional and governmental animal care committee (Ref. no. 54-2532.1-36/13, 55.2-2532-2-1121, Regierung von Unterfranken, Germany; University of Regensburg, Germany).

### Chronic in vitro electrical field stimulation of human induced pluripotent stem cell derived cardiomyocytes

Human iPSC–CM were differentiated from four healthy individuals by sequential targeting of the WNT pathway as described previously [12, 32, 65]. Following differentiation, purity and quality of iPSC–CM were determined by flow cytometry (~ 90% cardiac TNT +), cardiac immunofluorescence, morphology, and qPCR for cardiac sub-type marker (data not shown). Measurements were performed using 90-day-old iPSC–CM. To simulate persistent tachycardia, iPSC–CM cultures were subjected to chronic electrical field stimulation with 120 bpm for 24 h (early TACH) or 7 days (TACH). IPSC–CM paced with 60 bpm for 24 h or 7 days served as control groups (early CTRL or CTRL, respectively). The study was approved by the ethics committee of the University of Göttingen, Germany (Ref. no. 10/9/15).

### Transmission electron microscopy

LV specimens and iPSC–CM cultures for transmission electron microscopical analyses were prepared according to laboratory standard procedures. Transmission electron microscopy was performed by use of an EFTEM LEO912AB (Zeiss, Jena, Germany). Images were acquired with a 1 k × 1 k pixel side-entry mounted camera controlled by the iTEM software (OSIS, Muenster, Germany).

### Fluorescence and confocal microscopy

Cardiomyocyte cross-sectional area in LV and right ventricular (RV) tissue was quantified by Wheat Germ Agglutinin staining (Thermo Fisher Scientific, Waltham, MA, USA), connective tissue by Masson’s trichrome staining (Sigma-Aldrich, St. Louis, MO, USA). Apoptosis rate in LV and RV tissue was assessed by TUNEL staining (DeadEnd Colorimetric TUNEL System, Promega, Madison, WI, USA). Mitochondrial distribution was visualized by confocal microscopy (LSM 7 Pascal, Zeiss), staining LV and RV tissue with antibodies against HSP60 (ab59457, Abcam, Cambridge, UK) and N-Cadherin (AB0071-200, OriGene Technologies, Rockville, MD, USA).

### Pathway-focused transcriptomics

For pathway-focused gene expression analysis of LV in SHAM vs. TCM, custom-made PCR array kits (RT2 Profiler PCR Array, Qiagen, Venlo, the Netherlands) targeting mitochondrial metabolism and oxidative stress were employed.

### Mitochondrial and tissue redox state

Mitochondrial redox state NADH/NAD^+^ in rabbit LV was determined by a fluorometric assay kit (ab176723, Abcam). Tissue redox state in LV was assessed as the ratio of glutathione (GSH) to glutathione disulfide (GSSG), correcting for its dimeric state, by a previously described [63] colorimetric assay. Quantification of lipid peroxidation was performed using the ALDetect Lipid Peroxidation Assay Kit (Enzo Life Sciences, Farmingdale, NY, USA) to measure the concentration of malondialdehyde according to the manufacturer’s instructions.

### Metabolome analysis

Metabolite profile of rabbit LV tissue was analysed using liquid chromatography–high resolution mass spectrometry. After homogenization and extraction, SHAM and TCM samples were measured in a stratified randomized sequence with a Dionex Ultimate 3000 HPLC (Thermo Fisher Scientific) as previously described [83]. For additional evaluation of metabolic alterations in early disease, metabolomic profiling was repeated including ELVD samples using ultra-high performance liquid chromatography–high resolution mass spectrometry (UHPLC–HRMS) with a UHPLC Vanquish coupled to a QExactive mass spectrometer (Thermo Fisher Scientific). Metabolites were identified based on reference accurate mass and retention time. To correct for weight differences and technical variability, median normalization was performed. The complete data set is provided in Supplemental Datasheet 1 and 2.

### Acetylome analysis

For investigation of the mitochondrial acetylome in rabbit LV, sequential window acquisition of all theoretical mass spectra (SWATH–MS) analysis of isolated mitochondria was performed as described previously [31]. Mitochondrial proteome data were analysed with a focus on changes in the abundance of acetylated peptides. A library was built using UniProt_TrEMBL entries for rabbit (Version 2021_01) and ProteinPilot (v5.0, AB Sciex, Darmstadt, Germany). SWATH-runs were processed using PeakView (v2.2, AB Sciex). Label free data were exported, normalized to total intensity, and filtered either for acetyl-bearing peptides or pairs of modified and unmodified peptides.

### High-resolution respirometry

To evaluate mitochondrial respiration, permeabilised rabbit LV tissue, isolated mitochondria from LV tissue, and iPSC–CM cultures were subjected to high-resolution respirometry using a Oroboros-O2k oxygraph (Oroboros Instruments, Innsbruck, Austria). Applying substrate inhibitor titration protocols, oxidative phosphorylation capacity (OXPHOS) and electron transfer system capacity (ETS) were quantified [66, 77]. Respiration rates were normalized to wet tissue weight (whole tissue), protein content (isolated mitochondria) or photometrically determined citrate synthase activity (iPSC–CM). Hydrogen peroxide emission and membrane potential of isolated mitochondria were quantified simultaneously using Amplex UltraRed (Thermo Fisher Scientific) and TMRM (Sigma-Aldrich) [63].

### Mitochondrial calcium retention

Calcium retention in isolated mitochondria from rabbit LV tissue was quantified as described previously [63] with minor modifications. Mitochondria were preincubated in the absence or presence of cyclosporine A. After adding Calcium Green 5-N (Thermo Fisher Scientific), the assay was initiated by sequential additions of 10 µmol/L free Ca^2+^ every 2 min.

### Enzyme activities

Activity of isocitrate dehydrogenase type 2 (IDH), aconitase, and malate dehydrogenase (MDH) was determined by measuring absorption changes of NAD(P)H at 340 nm in reaction buffer containing the respective substrates.

### Flow cytometry for mitochondrial ROS emission, mitochondrial content, and apoptosis

To determine mitochondrial ROS emission (mitoROS), mitochondrial content, and apoptosis in iPSC–CM, cells were stained with MitoSOX Red, MitoTracker Green FM (both Thermo Fisher Scientific), and APC Annexin V (BioLegend, San Diego, CA, USA) and subjected to flow cytometry. For evaluation of mitoROS emission in the absence of cardiomyocyte contraction, the myosin II ATPase inhibitor blebbistatin 2,5 µM (Sigma-Aldrich) was added to the cell culture medium during electrical field stimulation.

### Western blot

Protein expression of oxidative phosphorylation complexes and key regulators of mitochondrial dynamics in rabbit LV was determined by western blot as described previously [5]. Details on the antibodies used are provided in the Supplementary Information.

### Serum high-sensitivity cardiac troponin T and natriuretic peptide measurements

High-sensitivity cardiac troponin T levels in rabbit serum samples were measured using an immunoassay (Roche Diagnostics, Rotkreuz, Switzerland) as previously described [23]. To evaluate natriuretic peptide levels as a surrogate of LV end-diastolic pressure [20], BNP gene expression was quantified in rabbit LV by real-time RT-PCR (QuantiTect SYBR Green, Qiagen). Tissue concentration of cGMP in rabbit LV was measured by a competitive enzyme immunoassay (cGMP ELISA kit, Cell Biolabs, San Diego, CA, USA).

### Statistical analysis

Data are shown as mean ± standard error of the mean (SEM), if not indicated otherwise. Differences between two groups in unpaired or paired design were tested for statistical significance by unpaired and paired two-sample *t* tests, respectively. Differences between more than two groups were tested by analysis of variance (ANOVA) with Tukey post-hoc tests. Data from mitochondrial calcium retention assays were analysed by two-way ANOVA. A two-sided *p* value < 0.05 was considered statistically significant. To determine effect size between two groups, Cohen’s *d* was calculated as the difference between the means divided by the pooled standard deviation. Differences in gene expression data were assessed using Student’s *t* test with a Bonferroni-adjusted significance level of *α* = 0.00030 to compensate for multiple testing. Metabolomics data were analysed calculating principal component analysis, analysis of variance, and multiple analysis of variance with pairwise post-hoc tests as detailed in the Supplementary Information. Statistical analyses were performed with Prism (v9.1.1, GraphPad Software, San Diego, CA, USA), R (v3.4.1, R Core Team, packages stats, FactoMineR, missMDA, nlme, lsmeans, readxl, openxlsx), and TIBCO Spotfire (v7.5.0, TIBCO, Palo Alto, USA).

## Results

### Tachypacing induces left ventricular non-fibrotic eccentric hypertrophy and severe systolic heart failure

Tachypacing induced increasing LV diameters and progressive systolic dysfunction from baseline via ELVD to TCM (Fig. 1B, C). All TCM animals showed signs of elevated LV end-diastolic pressure, such as enlarged left atria and increased BNP formation (Fig. 1D, E). Tissue concentration of cGMP declined and all TCM animals encountered severe heart failure syndrome, including pericardial and pleural effusion as well as ascites (1F-H). ELVD already entailed systolic dysfunction, but levels of high-sensitivity cardiac troponin T and size of cardiac myocytes were comparable to SHAM (Fig. 1I–L, P). Hypertrophy of cardiac myocytes was only present in the advanced stage disease, as indicated by increased cross-sectional area despite stable number of nuclei (Fig. 1I–L). Rate of apoptosis and serum levels of high-sensitivity troponin T were only increased in end-stage disease (Fig. 1M–P). Despite severe heart failure syndrome and increased apoptosis, neither LV hydroxyproline content nor Masson’s trichrome stain indicated increased fibrosis (Fig. 1Q–T). Together, TCM entails LV dilatation, eccentric hypertrophy, and clinical signs of severe heart failure syndrome, but no relevant fibrosis.

### Mitochondria increase in size and display a unique distribution pattern

**Fig. 2:**
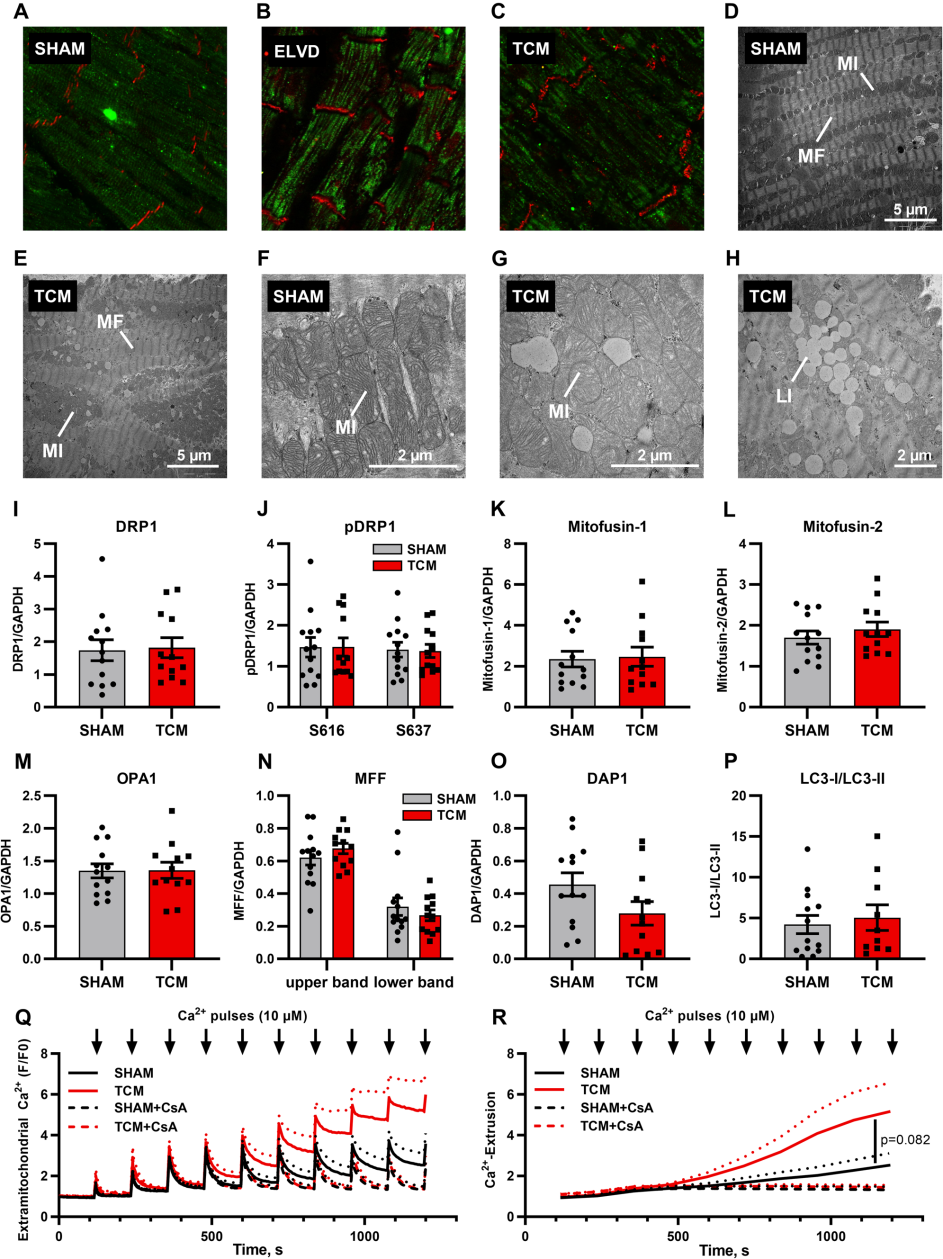
Tachycardiomyopathy entails disturbed mitochondrial distribution and morphology. In LV tissue of animals with advanced disease, the mitochondrial network was partially shifted from the sarcomeres to the intercalated discs (**A**–**E**). TCM animals showed giant mitochondria in TCM (**F, G**): LV tissue of TCM animals featured extensive perimitochondrial lipid droplets (**H**). Protein expression of key regulators of mitochondrial fusion, fission and mitophagy remained unchanged (**I**–**P**). Isolated mitochondria of TCM showed an earlier mitochondrial calcium release upon repetitive 10 µmol/l calcium pulses. Inhibition of the mitochondrial permeability transition pore by cyclosporine A (CsA) abolished the increased mitochondrial calcium release in TCM (**Q**, **R**). Data are shown as mean ± SEM. **R** *p* value for two-way ANOVA. **A**–**C** confocal microscopy with staining for HSP60 (green) and *N*-cadherin (red). **D**–**H** transmission electron microscopy. **Q** calcium retention capacity of isolated mitochondria exposed to repetitive calcium-pulses. Extramitochondrial calcium was monitored. Calcium extrusion was post-hoc calculated (**﻿R**) from original tracings (**﻿Q**). *CsA* cyclosporine A, *LI* lipid droplet, *MF* myofibril, *MI* mitochondrion

First, we investigated mitochondrial distribution and architecture. Comparing TCM to SHAM, the mitochondrial network was partially shifted from sarcomeres to the intercalated discs, as visualised by confocal microscopy and validated by electron microscopy (Fig. 2A–E). In ELVD, most mitochondria were still distributed in single layer rows between sarcomeres, only few cardiac myocytes showed mitochondrial enrichment at the intercalated discs (Fig. 2B). In TCM, increasing magnification of transmission electron microscopy visualised giant mitochondria (Fig. 2F, G). The augmented mitochondrial network at the intercalated discs included enriched perimitochondrial vacuoles of fat, which we saw exclusively in TCM specimens, but in none of the control samples (Fig. 2H).

Next, we set out to analyse potential mechanisms underlying the altered mitochondrial size and shape. Dynamin-related GTPases as main mitochondrial fission and fusion factors were analysed: the expression level of dynamin-related protein 1 (DRP1) as well as phosphorylation at its main sites, e.g., Ser616 and Ser637, did not change between SHAM and TCM (Fig. 2I, J; Supplementary Fig. 2). Similarly, the relative levels of mitofusins-1 (MFN1) and -2 (MFN2) and optic atrophy 1 (OPA1) remained stable between SHAM and TCM, as did the mitochondrial fission factor (MFF; Fig. 2K–N). Targeted transcriptomics entailed MFN1, OPA1, and FIS1, whose expression did not change in TCM (Supplementary Table 1). As mitochondrial fission and fusion factors did not explain formation of giant mitochondria, key regulators of mitophagy were evaluated next. However, neither the DAP1-level nor the ratio of LC3-I to LC3-II were altered in TCM (Fig. 2O, P), rendering a relevant role of disturbed mitophagy rather unlikely.

Finally, we were interested in the propensity for opening of the mitochondrial permeability transition pore (mPTP) in TCM. Thus, calcium retention capacity of isolated cardiac mitochondria from SHAM and TCM was measured by applying sequential calcium pulses while monitoring extramitochondrial calcium (Fig. 2Q, R). Each application of calcium led to a rapid increase in extramitochondrial calcium followed by a decay, mirroring mitochondrial calcium uptake. Repeated pulses led to slowed mitochondrial calcium intake and finally, calcium was released from mitochondria in SHAM as well as TCM. However, in TCM, mitochondrial calcium uptake slowed already after a few pulses and led to earlier mitochondrial calcium release. Cyclosporine A as an inhibitor of the mitochondrial permeability transition pore (mPTP) abolished the increased mitochondrial calcium release in TCM.

Taken together, the architecture of the mitochondrial network in TCM is characterised by a shift to the intercalated discs and enlarged mitochondria. In mitochondrial isolates, propensity for mPTP-opening increases.

### Mitochondria show severe depletion of tricarboxylic acid cycle intermediates and are shifted towards a more oxidised redox state in tachycardiomyopathy

Next, we set out to analyse whether the altered morphology of the precisely configured mitochondrial network is accompanied by changes in mitochondrial key functions, such as energy conversion, respiration, redox-balance, and ROS emission.

**Fig. 3:**
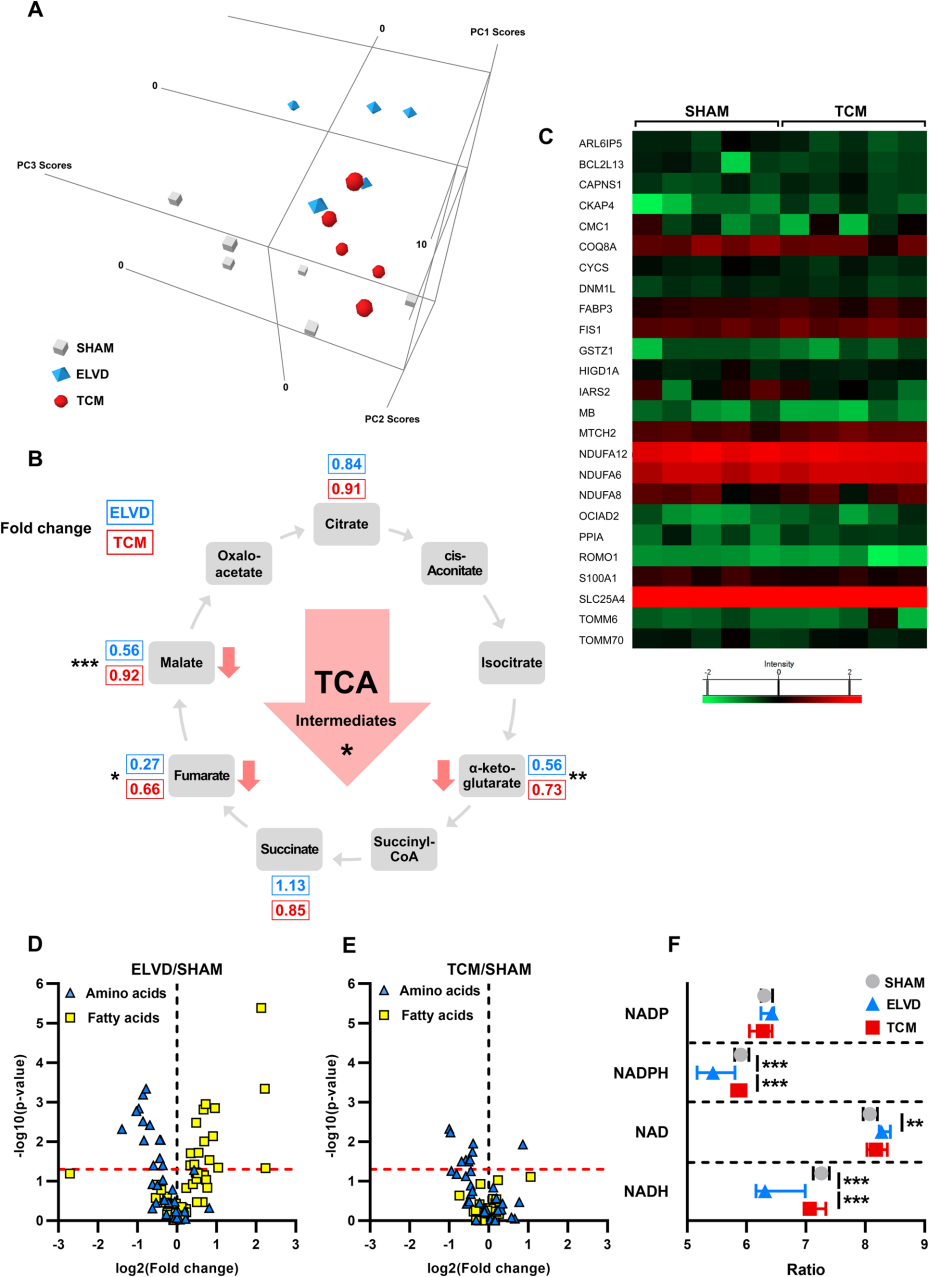
Tachycardiomyopathy is characterised by severe depletion of tricarboxylic acid cycle intermediates and emptying of the NADPH and NADH pool with a more pronounced effect in early disease than in heart failure state. Principal component analysis of metabolomics data of LV tissue clearly separated SHAM, ELVD and TCM (**A**). TCA intermediates were depleted even more in ELVD than in TCM (**B**). Acetylomics of isolated LV mitochondria based on SWATH–MS analysis remained without differences in the acetylation status of 25 identified peptides (**C**), indicating unchanged mitochondrial acetylome in tachycardiomyopathy. ELVD was characterised by diminished amino acids and increased fatty acids (**D**), being partially recovered in TCM (**E**). NADH and NADPH were decreased in disease, with a more pronounced loss in ELVD than in TCM (**F**). **p < 0.01, ***p < *0.001* for ANOVA. **B** fold change ELVD/SHAM (blue) and TCM/SHAM (red) of identified intermediates in metabolome analysis. *LV* left ventricular, *NAD(H)* nicotinamide adenine dinucleotide, *NADP(H)* nicotinamide adenine dinucleotide phosphate, *SWATH*–*MS* sequential window acquisition of all theoretical mass spectra, *TCA* tricarboxylic acid cycle

LV cellular metabolomes of SHAM, ELVD and TCM were analysed by liquid chromatography–high-resolution mass-spectrometry: 230 metabolites were consistently detected and subjected to further analysis (Supplementary Fig. 3). Principal component analysis clearly separated SHAM, ELVD, and TCM specimens into three distinct groups, stressing a strong biological regulation of metabolism in tachycardiomyopathy (Fig. 3A). It was significantly driven by metabolites belonging to eight pathways, revealed by multivariate analysis of variance (Table 1) and post-testing of metabolites (Supplementary Table 2) across CTRL, ELVD, and TCM. All of them affect mitochondrial function.

**Table 1:** Significantly altered metabolic pathways in multivariate analysis of variance of the metabolomic profile of SHAM and TCM

Pathway	Number of included metabolites	Pr(> *F*)
Electron transfer system	7	< 0.0001
Energy	10	0.034
Glutathione metabolism	7	0.019
Linoleic acid metabolism	9	0.002
Pentose phosphate pathway	3	0.002
Purine metabolism	3	0.002
Pyrimidine metabolism	10	0.055
Tricarboxylic acid cycle	7	0.024

*Pr(*> *F)* indicate results of multivariate analysis of variance

In detail, tricarboxylic acid cycle (TCA) metabolites were mainly depleted in ELVD and TCM with a stronger effect in ELVD (Fig. 3B). Looking at the influx side of the mitochondrial TCA, amino acid breakdown was diminished in ELVD, whereas fatty acids increased (Fig. 3D). Eventually, fatty-acid breakdown decreased to its initial level in TCM (Fig. 3E). Major catabolic pathways converge to acetyl-coenzyme A (acetyl-CoA), delivering acetyl groups to TCA. From SHAM to TCM, a remarkable decrease in acetyl-CoA occurred: it was down to 29% in TCM.

Concerning the outflow of TCA in terms of its link to oxidative phosphorylation, TCA reduces nicotinamide adenine dinucleotide (NAD^+^) to NADH, which delivers electrons to the electron transport system [63]. The depleted TCA metabolites in ELVD and TCM were in line with a decrease of the reduced form (NADH; Fig. 3F) and an increase of the oxidised state NAD^+^ in ELVD. NADH and nicotinamide adenine dinucleotide phosphate (NADPH) are in an equilibrium and form the mitochondrial pool of reduced pyridine nucleotides, on which oxidative phosphorylation and antioxidative capacity rely [63]. NADPH is mainly recovered by the pentose phosphate pathway, whose initial metabolites accumulated in ELVD, suggesting a downstream obstruction (Supplementary Fig. 4, Supplementary Table 3). Depleted TCA and obstructed pentose phosphate pathway were well in line with a decrease in NADPH (Fig. 3F). The drop in NADPH- and NADH-levels was more pronounced in ELVD with a slight recovery between ELVD and TCM. (Fig. 3F).

Whereas mitochondrial acetyl-CoA and TCA depletion pointed towards reduced mitochondrial transmembrane transport in line with previous reports [86], targeted transcriptomics of LV specimen including 39 genes of mitochondrial transport excluded a significant regulation of the investigated mitochondrial translocases (Supplementary Table 1).

Summing up, the metabolite profile of tachycardiomyopathy is characterised by a remarkable depletion of TCA metabolites and a heavily reduced acetyl-CoA-level. In addition, the dysfunction of TCA and pentose phosphate pathway is well in line with the decrease of NADH and NADPH in progressive tachycardiomyopathy. Together, mitochondrial depletion of TCA intermediates and acetyl-CoA as well as decreased NADH- and NADPH-levels represent the metabolite profile of tachycardiomyopathy.

### Mitochondrial acetylome remains stable in tachycardiomyopathy

Human heart failure resulting from ischaemic and dilated cardiomyopathy is characterised by hyperacetylated mitochondria [50], which are the consequence of decreased NADH/NAD^+^ ratio and a subsequently lowered activity of NAD^+^-dependent mitochondrial deacetylases [45]. The previously published increased NAD(P)^+^-levels in heart failure were particularly contradicted by our results in the early disease stage. Hence, we hypothesised that tachycardiomyopathy is devoid of mitochondrial hyperacetylation in contrast to published data on other cardiomyopathies. Acetylomics based on SWATH–mass spectrometry identified 25 acetylated peptides in isolated mitochondria of LV specimen derived from SHAM and TCM. None of these acetylated peptides showed a difference between the two groups (Fig. 3C). In summary, the mitochondrial acetylome remains stable in tachycardiomyopathy, which sets it apart from other cardiomyopathies.

### Mitochondria show mild respiratory dysfunction and a stable ROS emission in tachycardiomyopathy

**Fig. 4:**
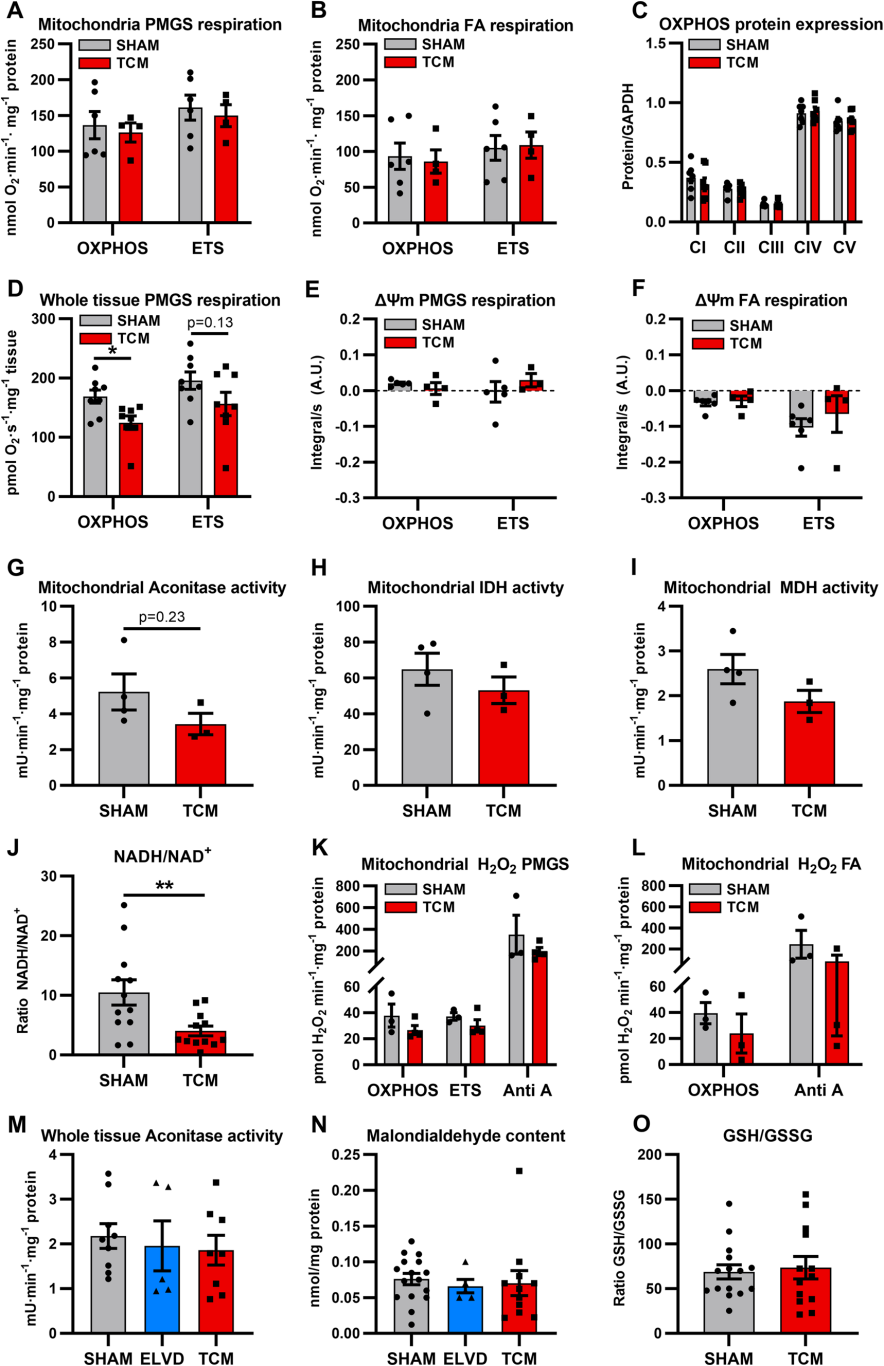
Tachycardiomyopathy implies mild respiratory dysfunction and stable ROS emission of mitochondria. Oxygen consumption of isolated mitochondria was similar for SHAM and TCM in the presence of pyruvate/malate/glutamate/succinate (**A** carbohydrate metabolism) and upon supplementation with fatty acids (**B** beta-oxidation). The expression levels of ETC complexes were equal in SHAM and TCM (**C**). In skinned fibres, OXPHOS declined (**D**). Simultaneous to oxygen consumption, mitochondrial membrane potential was measured and found to be similar for SHAM and TCM (**E**, **F**). Depletion of TCA metabolites was not explained by mitochondrial activities of aconitase, isocitrate dehydrogenase and malate dehydrogenase (**G–I**). Mitochondrial redox balance was shifted towards a more oxidised state (**J**). Neither pyruvate/malate/glutamate/succinate- nor fatty acid-respiration were accompanied by altered mitochondrial H_2_O_2_ emission in TCM (**K**, **L**). In LV tissue, mitochondrial aconitase activity and malondialdehyde content were similar for SHAM, ELVD, TCM (**M**, **N**). The ratio of the glutathione redox couple did not change (**O**). Data are shown as mean ± SEM. *p < 0.05, **p < *0.01* for *t* test. *ETS* electron transfer system capacity, *FA* fatty acid, *GSH* glutathione, *GSSG* glutathione disulphide, *IDH* isocitrate dehydrogenase, *MDH* malate dehydrogenase, *NAD* nicotinamide adenine dinucleotide, *OXPHOS* oxidative phosphorylation, *PMGS* pyruvate–malate–glutamate–succinate

Next, we assessed mitochondrial respiration and ROS formation. Oxygen consumption of isolated SHAM– and TCM–mitochondria was determined in the presence of pyruvate/malate/glutamate/succinate (for carbohydrate metabolism) and upon supplementation with fatty acids (for beta-oxidation). The maximum capacity to produce ATP (OXPHOS) and the maximum capacity of the electron transfer system (ETS) were equal in mitochondria isolated from SHAM and TCM (Fig. 4A, B). The complexes of the electron transport system were similarly expressed in SHAM and TCM (Fig. 4C). Next, we measured oxygen consumption of skinned fibres to estimate respiration of mitochondria embedded in their cardiac myocytes. Contrary to mitochondrial isolates, we detected impaired mitochondrial respiration in skinned fibres of TCM: OXPHOS declined significantly (Fig. 4D), whereas ETS was decreased by trend. The effect in OXPHOS decay exceeded ETS reduction (Cohen’s *d* 1.39 vs. 0.80). Together, TCM mitochondria showed an OXPHOS decay in skinned fibres, which was not fully explained by ETS function.

Simultaneous to oxygen consumption, mitochondrial membrane potential was measured using TMRM. It did not differ between SHAM and TCM (Fig. 4E, F). Considering the pronounced depletion of TCA metabolites encountered by our metabolome profiling, we set out to analyse main enzyme activities of TCA: mitochondrial activities of aconitase, isocitrate dehydrogenase, and malate dehydrogenase were similar in SHAM, ELVD, and TCM (Fig. 4G–I).

As the mitochondrial redox balance (NADH/NAD^+^) was shifted towards a more oxidised state (Fig. 4J), we were interested in mitochondrial ROS emission and employed a multi-faceted approach: in isolated mitochondria, H_2_O_2_ emission was measured by Amplex UltraRed. Nor pyruvate/malate/glutamate/succinate- neither fatty acid-respiration led to an increase in mitochondrial H_2_O_2_ emission in TCM (Fig. 4K, L). In LV tissue, mitochondrial aconitase activity and malondialdehyde content were similar at any time (SHAM, ELVD, TCM; Fig. 4M, N). The ratio of the glutathione redox couple did not change between SHAM and TCM (Fig. 4O). Together, our multi-faceted analyses did not reveal any evidence for relevant oxidative stress in TCM.

Considering the entire functional analyses, mitochondria showed mild respiratory dysfunction. In line with the results from metabolite profiling, TCA depletion is not due to mitochondrial respiration, membrane potential, or TCA enzyme activities. Finally, no evidence of oxidative stress was found in tachycardiomyopathy.

### Mitochondrial function in tachypaced human iPSC–CM

**Fig. 5:**
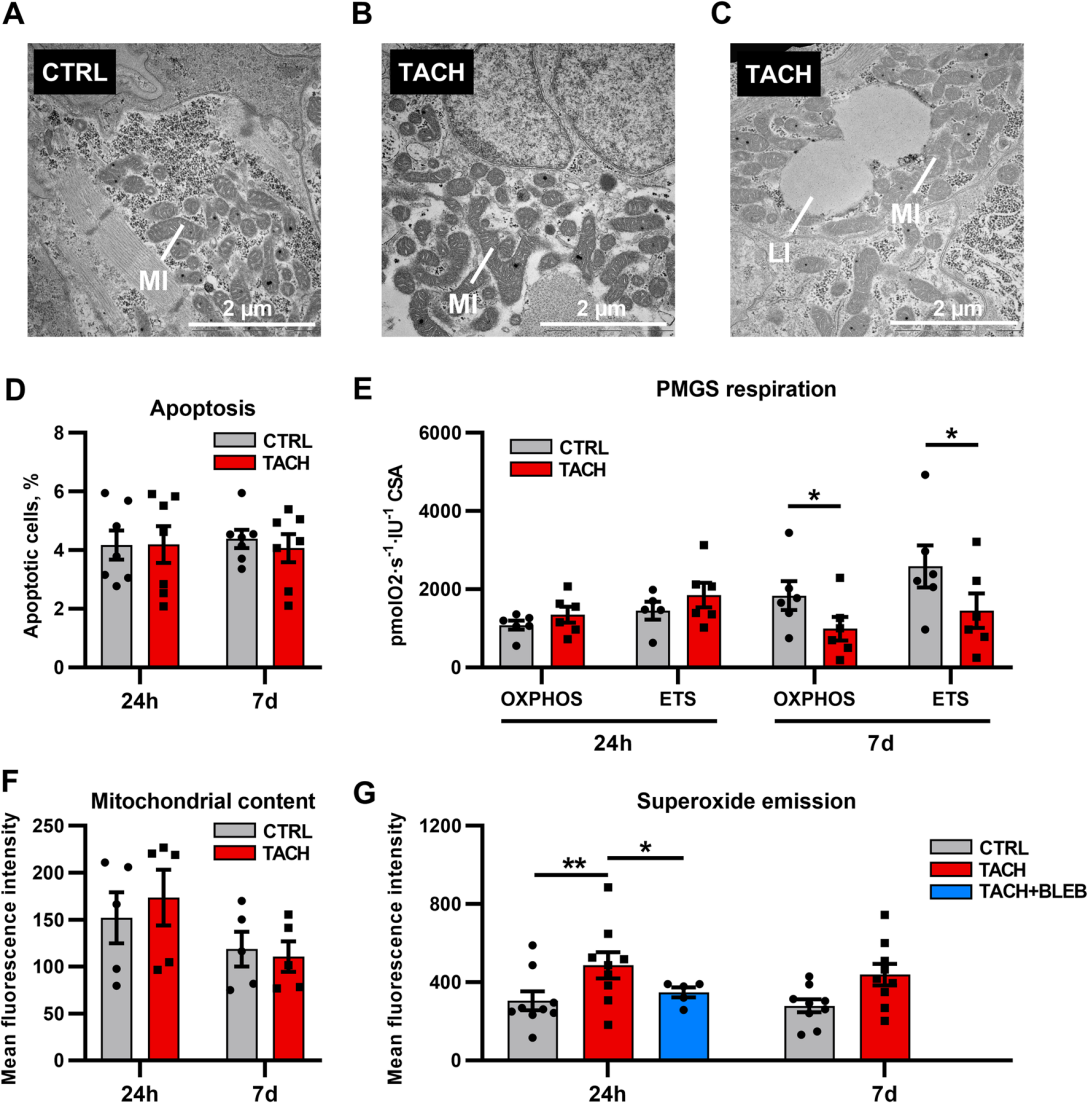
Mitochondrial function in iPSC–CM tachypaced for 1 and 7 days. Seven days of tachypacing lead to mitochondrial enlargement and pleomorphy. Furthermore, an increased number of lipid droplets was found compared to the control group (**A–C**). Apoptosis rate did not increase (**D**). Oxygen consumption remained stable after 24 h, but mildly decreased after 7 days (**E**). Mitochondrial content was unchanged after 24 h and 7 days (**F**). Mitochondrial ROS emission (MitoSOX) rose after 24 h, with inhibition of contraction by blebbistatin cancelling most of the effect (**G**). Data are shown as mean ± SEM. *p < 0.05, **p < 0.01 for paired *t* test CTRL vs. TACH and *t* test TACH vs. TACH + BLEB. **D** *n* = 7 differentiations of 3 donors. **E** *n* = 6 differentiations of 4 donors. **F** *n* = 5 differentiations of 2 donors. **G** *n* = 9 differentiations of 2 donors for CTRL and TACH, *n* = 4 differentiations of 1 donor for TACH + BLEB. **A**–**C** transmission electron microscopy. *BLEB* Blebbistatin, *CSA* citrate synthase activity, *ETS* electron transfer system capacity, *iPSC*–*CM* induced pluripotent stem cell cardiomyocyte, *LI* lipid droplet, *MI* mitochondrion, *OXPHOS* oxidative phosphorylation capacity, *PMGS* pyruvate–malate–glutamate–succinate

To validate our findings in a human model, we scrutinized mitochondrial function in a new model of tachypaced human iPSC–CM from healthy donors. After 7 days of tachypacing, mitochondrial enlargement in TACH–iPSC–CM resembled the giant mitochondria in the TCM animal model (Fig. 5A, B) as visualised by transmission electron microscopy. We also could find more lipid droplets in these cells compared to the control group (Fig. 5C).

Tachypacing for 7 days did not increase apoptosis compared to CTRL (Fig. 5D). After 24 h of tachypacing, mitochondrial respiratory capacity remained stable, ETS and OXPHOS decreased after 7 days (Fig. 5E). Reduced oxygen consumption was not explained by a difference in mitochondrial content (Fig. 5F), confirming a mild respiratory dysfunction in tachycardiomyopathy in a new human disease model. Mitochondrial ROS emission rose already after 24 h and remained consistently elevated in TACH–iPSC–CM compared to CTRL (Fig. 5G). However, chronic field stimulation under in-vitro conditions is known to increase ROS formation even in the absence of contraction [33], partially based on Faradaic side effects [51]. Thus, we added the myosin inhibitor blebbistatin [44] to TACH–iPSC–CM. The mitochondrial ROS emission of tachypaced iPSC–CM treated with blebbistatin was similar to CTRL (Fig. 5G). Unfortunately, supplementation to TACH–iPSC–CM for 7 days led to an overwhelming cytotoxicity, a known, ROS-independent complication of long-term treatment with blebbistatin [47, 82].

Taking the results derived from a novel human-based model of tachycardiomyopathy together, a mild increase in mitochondrial ROS emission was observed in iPSC–CM. However, relevant oxidative stress derived from mitochondria of beating iPSC–CM was ruled out, confirming the results of our animal model.

### Right ventricular pacing does not primarily induce right ventricular remodelling–sensitivity analysis

**Fig. 6:**
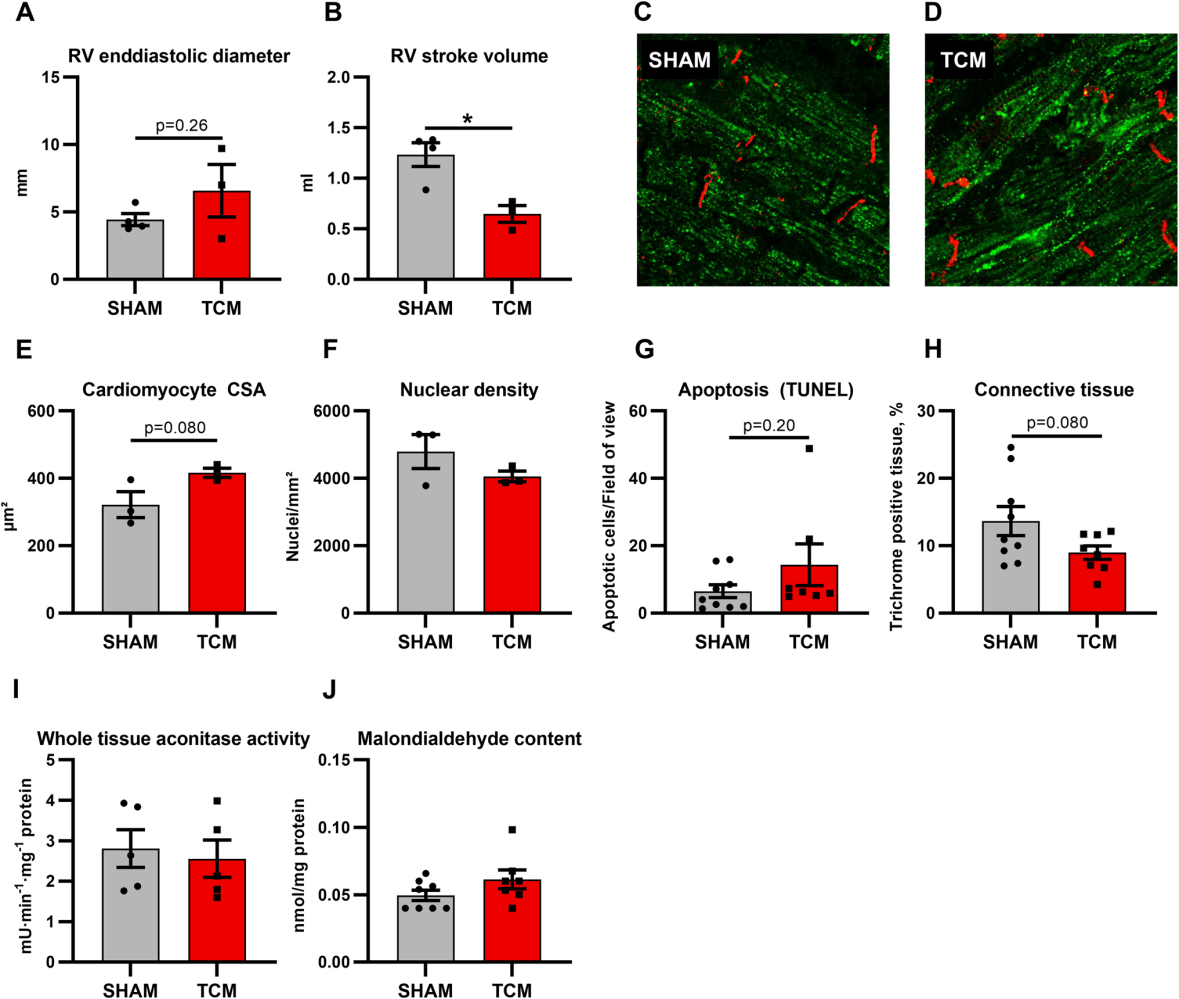
Tachypacing does not primarily induce right ventricular remodelling. After 30 days of tachypacing, echocardiography revealed a trend towards right ventricular dilatation (**A**) and decreased right ventricular stroke volume (**B**). Mitochondrial distribution in right ventricular cardiac myocytes was only subtly altered (**C, D**). Histology of right ventricular tissue showed a trend towards cardiomyocyte hypertrophy (**E, F**) and increased apoptosis (**G**) without fibrosis (**H**). Aconitase activity and malondialdehyde content remained unchanged (**I, J**). Thus, no evidence of oxidative stress was found. Data are shown as mean ± SEM. *p < 0.05 for t test. **C**, **D** confocal microscopy with staining for HSP60 (green) and *N*-cadherin (red). *CSA* cross-sectional area, *RV* right ventricular

Since previous work based on epicardial LV tachypacing pointed towards increased oxidative stress and fibrosis [35, 36], we wondered whether our opposing results might be a consequence of endocardial RV pacing in our model. Thus, the RV could be more affected than LV. To address this issue, we analysed RV specimens for sensitivity purpose (Fig. 6). Echocardiography visualised a trend towards RV dilatation (Fig. 6A). RV stroke volume decreased in TCM (Fig. 6B). Mitochondrial distribution in RV cardiac myocytes was barely changed in TCM (Fig. 6C, D). Cross-sectional area of RV cardiac myocytes tended to increase (Fig. 6E, F) and a trend towards elevated apoptotic rate was determined (Fig. 6G). Masson’s trichrome stain did not detect RV fibrosis in TCM (Fig. 6H). Regarding oxidative stress, neither aconitase activity nor malondialdehyde level, which serve as surrogate markers of acute and long-term ROS elevation, respectively, were increased (Fig. 6I, J). Together, quite similar processes occur to the left as the right ventricle in tachycardiomyopathy regarding hypertrophy, apoptosis, fibrosis, mitochondrial distribution, and oxidative stress.

## Discussion

**Fig. 7:**
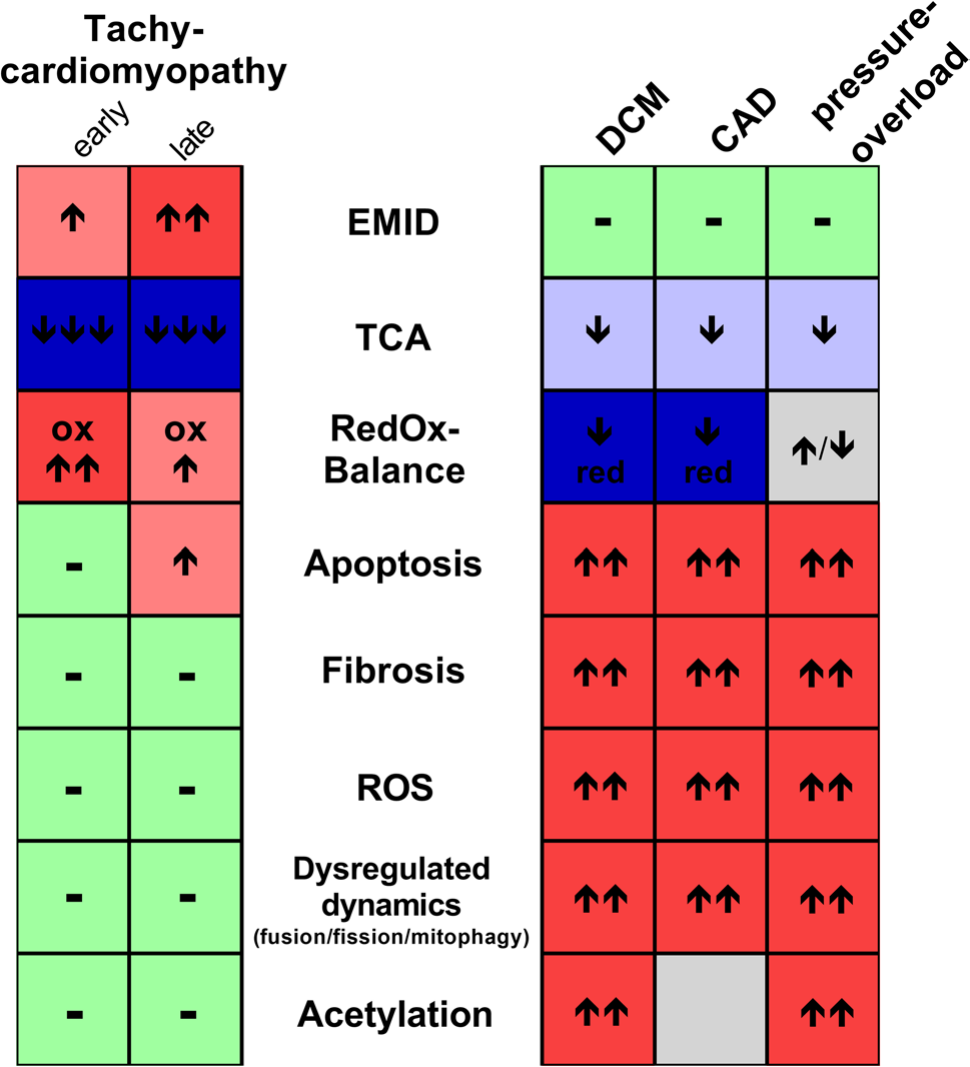
Mitochondrial functions in tachycardiomyopathy and other heart failure aetiologies. The two columns on the left side summarise our results on mitochondrial functions in tachycardiomyopathy. The three columns on the right side compile the current knowledge on main mitochondrial functions in dilated cardiomyopathy (DCM) as well as in heart failure due to coronary artery disease (CAD) or pressure-overload [1, 4, 7, 10, 14–16, 24–29, 37, 38, 41, 48–50, 52–55, 59, 60, 62–64, 69, 70, 72, 74, 76, 81, 85]. *EMID* enrichment of mitochondria at intercalated discs, *Redox balance* ratio of reduced to oxidised nicotinamide adenine dinucleotide, *ROS* reactive oxygen species, *TCA* intermediates of the tricarboxylic acid cycle

Our study analysed mitochondrial functions in tachycardiomyopathy. Despite severe systolic heart failure after 30 days of tachypacing, tachycardiomyopathy entailed only mild apoptosis and no fibrosis. The mitochondrial network was shifted to the intercalated discs, forming giant mitochondria. The metabolite profile of TCM was characterised by depletion of TCA-intermediates and emptying of the NADPH- and NADH-pool, with a more pronounced effect in early disease than in heart failure state. Depleted acetyl-CoA and a significant decrease in the NADH/NAD^+^-ratio were in line with a stable mitochondrial acetylome. In tachycardiomyopathy, mitochondria showed mild respiratory dysfunction. No evidence of relevant oxidative stress was detected in early as well as end-stage tachycardiomyopathy. Our key findings of mildly decreased oxidative respiratory capacity and absence of evidence of mitoROS-induced oxidative stress were validated in a new human-based iPSC–CM model. Therefore, our results may be translated to human disease. Together, our systems medicine approach describes mitochondrial characteristics of tachycardiomyopathy, which differ from hitherto published signs of other heart failure aetiologies (Fig. 7).

First, several advantages and limitations of our disease models should be taken into further consideration: rabbits mimic human physiology in tachycardia to a higher degree than small rodents, as humans and rabbits can raise their heart rate about 130% and more, whereas mice are limited to a maximum heart rate increase of 30% [17, 43, 79]. In addition, excitation–contraction coupling and energy supply in cardiac myocytes of rabbits resembles human physiology to a significant larger extend than, e.g., in mice [6, 57, 67], as validated by recent computational approaches [58]. In conclusion, the rapid-pacing model in rabbits provides several advantages regarding translation to human disease. However, right ventricular pacing implies a cardiac conduction abnormality, resembling left bundle branch block. Thus, our data derived from the rabbit model might be influenced by pacing-induced LV conduction delay [2]. However, the key findings were also seen in tachypaced iPSC–CM, which are not biased by intraventricular conduction.

Indeed, our data mirror ultrastructural remodelling in human tachycardiomyopathy in terms of absent fibrosis, as far as it is known in human disease at the moment: there is consistent literature reported by clinical trials as well as imaging studies supporting absence or only mild occurrence of fibrosis in tachycardiomyopathy [30, 40, 70, 78]. The enrichment of mitochondria at intercalated discs (EMID sign) was first revealed by confocal microscopy analysing endomyocardial biopsies of tachycardiomyopathy patients [59]. It was similarly seen in our animal model. We additionally performed transmission electron microscopy in specimen of TCM as well as in human-based TACH–iPSC–CM. In both models, the EMID sign corresponds to giant mitochondria augmented at the intercalated discs. We did not find any evidence that alterations in mitochondrial key factors of fission, fusion or mitophagy could explain mitochondrial enlargement in tachycardiomyopathy. The observed increased propensity for mPTP-opening in mitochondrial isolates might give a hint towards swelling underlying the mitochondrial enlargement. However, extreme caution is required when interpreting this finding, as there are severe methodical limitations to acknowledge. Increased mPTP-opening could also have resulted from an overall cardiomyocyte injury, yielding a suboptimal fraction of mitochondria during the isolation process. Thus, the mechanism of the pronounced mitochondrial enlargement still remains elusive at this stage. Future studies are warranted to settle this question.

The mitochondrial metabolite profile of TCM is characterised by a severe depletion of TCA intermediates, occurring already in early disease and being the first mitochondrial sign of progressive tachycardiomyopathy. Of particular note, myocardial concentration of acetyl-CoA dropped markedly in TCM, which is unique to tachycardiomyopathy, has not been detected in other heart failure aetiologies until now and contrasts dilated cardiomyopathy [8]. TCA activity provides reduced NADH which delivers electrons to the respiratory chain by being oxidised to NAD^+^. The level of the reduced form of NADH is connected to reduced NADPH via mitochondrial transhydrogenase and other pathways [63, 73]. NADPH is a mainstay for the antioxidative capacity of mitochondria [18]. Apart from TCA, pentose phosphate pathway can directly recover NADPH. However, the pentose phosphate pathway was nearly shut down in ELVD. With a depletion of TCA intermediates and a failing pentose phosphate pathway, the ratio of NAD(P)H to NAD(P)^+^ dropped, signifying a considerably oxidative mitochondrial state in tachycardiomyopathy. Opposed to our findings in tachycardiomyopathy, NADH/NAD^+^-ratio increases in afterload-induced and dilated cardiomyopathy [24, 50]. Of note, there are conflicting data on NAD(P)H/NAD(P)^+^-balance in dissimilar heart failure models which might result from divergent involvement of metabolism in different heart failure aetiologies [9]. Indeed, our study is the first to report a decreased NADH/NAD^+^ ratio in tachycardiomyopathy. This distinguishes tachycardiomyopathy from dilated cardiomyopathy and afterload-induced hypertrophy, which are characterised by an elevated NADH/NAD^+^ ratio.

The shift towards a more oxidised mitochondrial NADH/NAD^+^-balance in tachycardiomyopathy corresponds to a favourable effect on mitochondrial protein acetylation: in other heart failure aetiologies, the pro-reductive mitochondrial redox-balance, characterised by increased NADH and diminished NAD^+^-levels, inhibits mitochondrial NAD^+^-dependent deacetylases, e.g., sirtuin 3 [45]. Thus, the antioxidative and antihypertrophic effects of the NAD^+^-dependent sirtuins are hampered and the mitochondrial proteome becomes hyperacetylated [45, 50, 71, 80]. Indeed, supplementing an NAD^+^-precursor oxidises the reduced NADH/NAD^+^-balance and ameliorates progression to heart failure in preclinical mouse models [50]. Apart from mitochondrial NADH/NAD^+^-concentrations, the elevated concentration of acetyl-CoA in heart failure may additionally contribute to hyperacetylated mitochondria, as it provides acetyl-groups for acetylation [8, 9]. Together, hyperacetylation of mitochondrial proteins is a hallmark of mitochondrial remodelling in systolic heart failure [9], which has also been found consistently in specimen of human end-stage dilated cardiomyopathy by several research groups [37, 50]. In our study, tachycardiomyopathy diverges from dilated cardiomyopathy in as much as acetyl-CoA is depleted and the mitochondrial NADH/NAD^+^-balance shifted towards NAD^+^ due to a remarkable depletion of TCA intermediates. Hence, we may be first to report on a stable mitochondrial acetylome in a heart failure phenotype.

Whereas mitochondrial catabolism and NADH/NAD^+^-balance were severely disturbed, oxidative phosphorylation capacity was not diminished during respirometry of isolated mitochondria, which was performed under controlled conditions and saturating concentrations of oxygen and substrates (pyruvate/malate/glutamate/succinate or fatty acids, respectively). Thus, a relevant limitation of mitochondrial functions by its electron transport system per se does not seem plausible. However, oxidative phosphorylation in skinned fibres was reduced contrary to mitochondrial isolates. Whereas isolated mitochondria lose intracellular interaction and their three-dimensional structure degenerates to spherical organelles [68], permeabilization of fibres maintains mitochondrial morphology and their interaction with other organelles and the cytoskeleton [3, 13]. Thus, proper function of mitochondrial isolates and impaired respiration in skinned fibres may point towards issues due to mitochondrial structure or the mitochondrial–cytosolic crosstalk, which remains to be investigated in the future.

Since the antioxidative capacity of mitochondria is based upon their ability to restore reduced NADPH [18, 84] and previous studies reported on oxidative stress in tachycardiomyopathy as recently reviewed [34], we were further interested in mitochondrial ROS emission. Against expectations, in neither our animal nor iPSC–CM-model evidence for oxidative stress was detectable. The lack of oxidative stress in LV tissue was particularly striking, given the severe heart failure symptoms in the TCM animals (e.g., pericardial and pleural effusion, ascites and cachexia) in our present and earlier studies [21, 22]. In addition, oxidative stress has been established as a driver of heart failure disease during the past two decades in pressure-overload and ischemic heart disease as well as dilated cardiomyopathy [34, 41, 48, 53, 54]. However, it is tempting to speculate whether the remarkable absence of evidence for oxidative stress may be associated with the lack of signs of irreversible ROS-damage, namely, fibrosis, in tachycardiomyopathy.

Together, the described profile of mitochondrial dysfunctions sets the phenotype of tachycardiomyopathy apart from other aetiologies of systolic heart failure (Fig. 7). However, particular caution is needed when translating these mitochondrial alterations to contractile function. Though mitochondrial substrate depletion and the drop in NADH- and NADPH-levels in ELVD preceded the onset of severe systolic failure in the animal model, our data provide just evidence of phenomenological associations. It remains elusive whether mitochondrial derangements are primary determinants of tachypacing-induced systolic dysfunction. Room is left to the possibility that contractility is altered independently of mitochondrial alterations.

## Conclusions

Tachycardiomyopathy entails a distinct pattern of mitochondrial dysfunctions characterized by enlarged mitochondria shifted towards the intercalated discs, depletion of TCA substrates, and a shift of the mitochondrial redox balance towards a more oxidised state. However, there is no evidence for oxidative stress or relevant fibrosis. These findings contrast characteristic signs of previously reported heart failure aetiologies, such as ischemic, increased afterload, hereditary, and toxic dilated cardiomyopathy, which rather feature increases in NADH and oxidative stress as well as fibrosis and hyperacetylation of the mitochondrial proteome.

## Supplementary Information

Below is the link to the electronic supplementary material.

Supplementary file1 (PDF 7641 KB)

Supplementary file2 (XLSX 29504 KB)

Supplementary file3 (XLSX 22887 KB)
