# Supplementary material for: Tachycardiomyopathy entails a dysfunctional pattern of interrelated mitochondrial functions
Source: Basic Res Cardiol. 2022 Sep 6;117(1):45. doi: 10.1007/s00395-022-00949-0 (PMC9448689; doi:10.1007/s00395-022-00949-0)
Supplement: Supplementary file 1 — Supplementary file1 (PDF 7641 KB) [file 395_2022_949_MOESM1_ESM.pdf]

Tachycardiomyopathy entails a dysfunctional pattern of interrelated mitochondrial functions

Michael G. Paulus, Kathrin Renner, Alexander G. Nickel, Christoph Brochhausen, Katharina Limm, Elmar Zügner, Maria J. Baier, Steffen Pabel, Stefan Wallner, Christoph Birner, Andreas Luchner, Christoph Magnes, Peter J. Oefner, Klaus J. Stark, Stefan Wagner, Christoph Maack, Lars S. Maier, Katrin Streckfuss-Bömeke, Samuel Sossalla, Alexander Dietl

Basic Research in Cardiology

Supplementary Information

Corresponding author:
Alexander Dietl, MD
Department of Internal Medicine II, University Hospital Regensburg, Germany
E-mail: alexander.dietl@ukr.de

Supplementary Methods

Animal model of tachycardiomyopathy

For in vivo investigation of tachycardiomyopathy, an animal model was used as previously described [1]. Male New Zealand White rabbits were housed under standard conditions with regular, unrestricted diet. For pacemaker implantation, anaesthesia was induced by xylazine 5 mg/kg and ketamine 50 mg/kg i.m. and maintained by continuous i.v. administration (xylazine 1.5 mg/kg and ketamine 15 mg/kg) under close monitoring of the animal's vital signs. A custom-made, 2 Fr. unipolar pacemaker lead (DMTpe, Nufringen, Germany) was inserted into the right internal jugular vein and fixed into the right ventricular apex under fluoroscopic guidance. A programmable cardiac pacemaker (Advisa DR MRI SureScan, Medtronic, Minneapolis, MN, USA) was implanted subcutaneously into the right abdominal wall and connected to the subcutaneously tunnelled pacemaker lead. In the early post-surgery period, carprofen 4 mg/kg and enrofloxacin 5 mg/kg were administered subcutaneously for analgesia and antibiotic prophylaxis. After recovery of at least 10 days, incremental tachypacing was conducted by programming a V00 mode with a cycle length of 182 ms (330 bpm) for ten days, followed by 167 ms (360 bpm) for ten days and 158 ms (380 bpm) for further ten days. After completion of tachypacing, echocardiographic assessment was performed under moderate sedation (xylazine 1 mg/kg i.m. and ketamine 10 g/kg i.m.) and temporarily intermitted pacing using a Sonos 5500 equipped with a 12MHz transducer (Philips Healthcare, Amsterdam, Netherlands). Left ventricular end diastolic diameter (LVEDD), end systolic diameter (LVESD), left atrial diameter, and right ventricular end diastolic diameter were measured by two-dimensionally guided M-Mode in the parasternal long axis. Systolic function was determined by calculating fractional shortening (FS) as FS = (LVEDD-LVESD)/LVEDD. Right ventricular stroke volume was determined by measuring right ventricular outflow tract diameter in B-Mode and outflow velocity time integral by pulsed-wave doppler interrogation. Afterwards, animals were killed, and organs harvested for further analysis. A total of 24 rabbits underwent tachypacing for 30 days (tachycardiomyopathy, TCM), while 26 sham-operated rabbits served as a control group (SHAM). To investigate early disease, 11 animals underwent tachypacing for a shortened period of 10 days (early left ventricular dysfunction, ELVD). The animal study was approved by the institutional and governmental animal care committee (ref. no. 54-

2532.1-36/13, 55.2-2532-2-1121, Regierung von Unterfranken, Germany; University of Regensburg, Germany).

Chronic in vitro electrical field stimulation of human induced pluripotent stem cell cardiomyocytes

Human induced pluripotent stem cell cardiomyocytes (iPSC-CM) were differentiated from four healthy individuals as previously described [2]. Somatic cells were reprogrammed using nonintegrating systems, followed by differentiation into iPSC-CM by Wnt modulation and metabolic selection. Purity of iPSC-CM was confirmed by flow cytometry (~90% cardiac TNT+), cardiac immunofluorescence, morphology, and qPCR for cardiac sub-type marker (data not shown). After cultivation for 90 days, cells were transferred into 6-well plates equipped with C-Dish carbon electrodes connected to a C-Pace EM electrical stimulator (both IonOptix, Westwood, MA, USA). To simulate persistent tachycardia, iPSC-CM cultures were subjected to chronic electrical field stimulation with 120 bpm for 24 hours (early TACH) or seven days (TACH). IPSC-CM paced with 60 bpm for 24 hours or seven days served as control groups (early CTRL or CTRL, respectively). Effective capture during field stimulation was verified by light microscopic visualization of cell contraction. To account for variation between cell differentiation experiments, chronic in vitro stimulation of iPSC-CM and subsequent experiments were carried out in a paired design. The study was approved by the ethical committee of the University of Göttingen, Germany (ref. no. 10/9/15).

Transmission Electron microscopy

Transmission Electron Microscopy (TEM) was performed to analyse ultrastructural changes in cardiomyocytes with focus on mitochondrial distribution in the cell. For this purpose, rabbit left ventricular (LV) tissue samples were fixed in Karnovsky-fixative (0.1M cacodylate-buffer with 2.5% glutaraldehyde and 2% paraformaldehyde) for at least 48 hours, followed by post-fixation with 1% osmium tetroxide at pH 7.3. Samples were then dehydrated in graded ethanol, embedded in EMbed-812 epoxy resin (Science Services, Munich, Germany) and finally polymerized for 48 hours at 60°C into an EPON block. Semithin sections of 0.75µm thickness were cut and stained with toluidine blue and basic fuchsine. After selection of appropriate

areas of interest, the EPON block was trimmed, and ultrathin sections (80nm thickness) were cut on a Reichert Ultracut-S ultramicrotome (Leica, Wetzlar, Germany). Sections were mounted on grids and stained with aqueous 2% uranyl acetate and lead citrate solution for 10 min each. IPSC-CM samples were cultured in 6-well-plates. The cell culture medium was discarded and replaced with Karnovsky-fixative for at least 6 hours. Afterwards, the cells were scraped and pelletized at 200g. The cell pellets were then enclosed with cytoblock (Epredia, Portsmouth, NH, USA), then with 4% low melting agarose (Thermo Fisher Scientific). The further handling was the same as for the tissue samples. The sections were analysed by means of a LEO912AB electron microscope (Zeiss, Jena, Germany) operated at 100kV. Images were taken by a side-mounted 2k x 2k-CCD-camera (TRS, Moorenweis, Germany).

Fluorescence and confocal microscopy

For fluorescence microscopy, paraffinized rabbit LV and right ventricular (RV) tissue was deparaffinized and stained with Wheat Germ Agglutinin Alexa Fluor 594 Conjugate (W11262, Thermo Fisher Scientific, Waltham, MA, USA), DAPI (D1306, Thermo Fisher Scientific), and Masson's trichrome (HT15-1KT, Sigma-Aldrich, St. Louis, MO, USA). TUNEL-staining was performed using the DeadEnd Colorimetric TUNEL System (Promega, Madison, WI, USA) according to the manufacturer's instructions. Images were acquired by an Axio Observer Z1 equipped with an Axiocam MRm (Zeiss). Cell cross-sectional area (CSA) of cardiomyocytes was determined by manually tracing cell borders using HistoQuest (v.6.0, TissueGnostics, Vienna, Austria). Nuclei density was quantified by manual counting using ImageJ (v.1.53g). Proportion of trichrome positive tissue was quantified automatically by ImageJ. For confocal microscopy, paraffinized rabbit LV and RV tissue was deparaffinized and stained with antibodies against HSP60 (ab59457, Abcam, Cambridge, UK) and N-Cadherin (AB0071-200, OriGene Technologies, Rockville, MD, USA). As secondary antibodies, Donkey anti-Goat IgG Alexa Fluor 568 (A-11057, Thermo Fisher Scientific) and Donkey anti-Mouse IgG Alexa Fluor 488 (A-21202, Thermo Fisher Scientific) were applied. Confocal laser scanning images were acquired using a LSM 7 Pascal (Zeiss).

Hydroxyproline content

To evaluate collagen content in rabbit LV, hydroxyproline concentration in homogenized tissue was quantified using a commercially available colorimetric assay kit (ab222941, Abcam) according to the manufacturer's instructions.

Pathway-focused transcriptomics

mRNA was extracted from homogenized rabbit LV tissue using TRIzol reagent (Thermo Fisher Scientific) and the RNeasy Mini Kit (Qiagen, Venlo, Netherlands) according to the manufacturer's protocols. RNA yield was measured by spectrophotometry at 260 nm with a NanoDrop 2000c (Thermo Fisher Scientific). cDNA synthesis was performed using the RT^2 First Strand Kit (Qiagen). For pathway-focused gene expression analysis, commercially available PCR array kits (RT^2 Profiler PCR Array, Qiagen) targeting mitochondrial metabolism (PANZ-087Z) and oxidative stress (PANZ-065Z) were customized for rabbits and run in duplicate according to the manufacturer's instructions. All PCR reactions were performed with a ViiA7 Real-Time PCR system (Thermo Fisher Scientific). Analysis of gene expression data was conducted using the GeneGlobe Data Analysis Center (https://geneglobe.qiagen.com/jp/analyze, accessed 12/2020, Qiagen). Differences in gene expression were assessed using Student's t-test with a Bonferroni-adjusted significance level of $\alpha=0.00030$ to compensate for multiple testing.

Mitochondrial and tissue redox state

To determine mitochondrial redox state NADH/NAD^+ in rabbit LV tissue, a commercially available fluorometric assay kit (ab176723, Abcam) was used according to the manufacturer's instructions. Tissue redox state GSH/GSSG in rabbit LV was quantified as previously described [3]. In brief, total glutathione was determined in homogenized tissue by measuring glutathione-dependent reduction of DTNB at 412 nm. For measuring GSSG, tissue was homogenized in the presence of 3 mM 1-methyl-2-vinylpyridine for derivatization of GSH before determining reduction of DTNB. GSH content was calculated as the difference between total glutathione minus the values obtained for GSSG. Quantification of lipid peroxidation was

performed using the ALDetect Lipid Peroxidation Assay Kit (Enzo Life Sciences, Farmingdale, NY, USA) to measure the concentration of malondialdehyde according to the manufacturer's instructions. All colorimetric measurements were performed with an Infinite M200 Pro microplate reader (Tecan Group, Männedorf, Switzerland).

High-resolution respirometry of whole tissue and iPSC-CM

Preparation of permeabilised rabbit LV myocardium

Two to six mg of fresh rabbit LV myocardium were immediately placed in ice cold BIOPS, a preserving solution containing (in mM) 2.77 CaK_2EGTA, 7.23 K_2EGTA, 5.77 Na_2ATP, 6.56 $MgCl_2$, 20 taurine, 50 MES, 15 Na_2phosphocreatine, 20 imidazole, and 0.5 DTT adjusted to pH 7.1. Fibres were mechanically separated using pointed forceps in a petri-dish in BIOPS on ice, permeabilization was controlled by microscopy. Samples were subsequently permeabilised by gentle agitation for 30 min at 4°C in BIOPS containing 50 µg/mL of saponin followed by a washing step in MiR05 for 10 min at 4°C by gentle agitation. Afterwards, wet weight of the biopsies was determined and samples were immediately transferred to the oxygraph chambers for measurement of mitochondrial respiration.

Preparation of iPSC-CM cultures

$2.5*10^6$ cells/ml were harvested, resuspended in mitochondrial medium MiR05, and immediately transferred to the oxygraph chambers for determination of mitochondrial respiration.

Determination of mitochondrial respiration

Activity of the respiratory system was analysed in a two-channel titration injection respirometer (Oxygraph-2k, Oroboros, Innsbruck, Austria) at 37°C. In principle, the same protocol was applied to iPSC-CM cultures and to myocardial biopsies; however, cultured cells were measured under normoxic conditions from air saturation to 50 µM, whereas for cardiac muscle an oxygen range from 400 µM to 200 µM was used to avoid oxygen limitation due to diffusion. Oxygen levels were increased by the addition of H_2O_2 to a catalase (280U/ml) containing MiR05. The substrate inhibitor titration protocol (SUIT) was only in the beginning slightly different between cultured cells and cardiac muscle biopsies. In iPSC-CM, after a

stabilization phase of 25 min, ROUTINE respiration of intact cells was measured, subsequently malate was added, and the plasma membrane was permeabilized with digitonin (16.2 µM). For myocardial biopsies, malate was added immediately after fibres (already saponin permeabilized) had been transferred to the Oxygraph chambers followed by a stabilization phase of 10 to 20 min. In both set-ups, complex I activity was stimulated by the following substrates: pyruvate (5 mM), ADP (5 mM), and glutamate (10 mM). Capacity of the oxidative phosphorylation system was determined by convergent electron flow through complex I and II after addition of succinate (10 mM). Subsequently, capacity of the electron transport system was measured by uncoupling with p-trifluoromethoxy carbonyl cyanide phenyl hydrazone (FCCP, 1.5 µM), injected stepwise to avoid inhibitory effects [33]. After rotenone addition (0.5 µM), maximum capacity of complex II was measured. Finally, residual oxygen consumption was determined after myxothiazol addition (0.5 µM).

Quality of muscle fibre preparation based on integrity of the outer mitochondrial membrane was checked by the addition of cytochrome c (10 µM). Since cytochrome c had no stimulatory effect, integrity of the outer mitochondrial membrane could be concluded.

Determination of citrate synthase activity

Activity of the mitochondrial marker enzyme citrate synthase (CS) was determined in iPSC-CM cultures. After finishing the SUIT protocol, the total volume of medium containing suspended iPSC-CM was taken directly from the oxygraph chamber, shock frozen, and stored at -80°C. After thawing, specific CS activity ($IU*ml^{-1}$) in the samples was quantified photometrically measuring the conversion of DTNB to TNB at 412 nm coupled to the CS-catalysed reaction of acetyl-CoA and oxalacetate to citrate. For photometry, a NanoDrop 2000c (ThermoFisher Scientific) was used.

Normalization of respiration rates

Respiration rates were calculated as the time derivative of oxygen concentration per chamber volume ($pmol*s^{-1}*ml^{-1}$). For heart muscle fibres, respiration rates were normalized to wet tissue weight ($pmol*s^{-1}*mg^{-1}$). For iPSC-CM, respiration was normalized to CS activity ($pmol*s^{-1}*IU^{-1}$).

High-resolution respirometry of isolated mitochondria

Freshly isolated cardiac mitochondria from rabbit LV were used for high-resolution respirometry measurements that were performed as described previously [44]. Oxygen consumption was assayed at 37°C with an Oxygraph-2k high-resolution respirometer and DatLab software was used for data acquisition and analysis (Oroboros Instruments). Two mitochondrial respiration protocols were used to quantify oxygen consumption rate upon supplementation of pyruvate and glutamate (for carbohydrate metabolism) or fatty acids (for β-oxidation) as a fuel. In the "carbohydrate" protocol, measurements of complex I and II activity were performed with 5 mM each of pyruvate, glutamate, malate, and 10 mM succinate. The metabolites were added as reduced substrates after initially recording residual oxygen consumption resulting in leak respiration, followed by increasing concentrations of ADP (0.03, 0.1, 0.3, 1 mM). In the "fatty acid" protocol, respiration was measured using 1 mM carnitine, 3 mM malate, 10 µM palmitoyl-CoA, and 10 µM oleoyl-L-carnitine. Finally, oxygen consumption coupled to ADP phosphorylation was inhibited by adding 1.25 µM oligomycin followed by titration with 10 µM DNP to determine ETS capacity. Mitochondrial membrane potential was simultaneously probed using 1 µM TMRM and Smart Fluo-Sensor Green as described before [4].

Mitochondrial hydrogen peroxide emission in rabbit left ventricular tissue

H_2O_2 emission from isolated mitochondria was measured with Amplex UltraRed (Thermo Fisher Scientific), a sensitive and H_2O_2-specific indicator that is converted to the fluorescent molecule resorufin (λexc 535 nm; λem 595 nm) upon reaction with H_2O_2. Oxidation of Amplex UltraRed by H_2O_2 is catalysed by horseradish peroxidase (HRP). The assay was performed at 37°C using an Oxygraph-2k high-resolution respirometer (Oroboros Instruments) with a Smart Fluo-Sensor Green-Unit as previously described [5].

Mitochondrial calcium retention

Mitochondrial calcium retention in isolated mitochondria from rabbit LV tissue was quantified as described previously [3] with minor modifications using a fluorescence plate reader (Infinite

M200Pro, Tecan Group) with an automated injection option. All experiments were conducted in 200 µl of buffer containing (in mM) 120 KCl, 70 mannitol, 25 saccharose, 20 HEPES, 5 KH_2PO_4, and 0.02 EGTA, with 165 µg of mitochondria per well in duplicates, respectively (excitation: 503 nm; emission: 535 nm). Mitochondria were preincubated with 5 mM K-glutamate/2.5 mM malate in the absence or presence of cyclosporine A (2 µM) for 5 min. 1 µM of Calcium Green 5-N (Thermo Fisher Scientific) was added before starting the measurement, and the assay was initiated by sequential additions of 10 µM free Ca^{2+} every 2 minutes per well.

Enzyme activities

All enzyme activities were determined by measuring absorption changes of NAD(P)H every minute at 340 nm in a total volume of 200 µl reaction buffer at 37°C after one freeze/thaw cycle to break mitochondria. For isocitrate dehydrogenase type 2 activity, 20 µg of mitochondria or cytosol was supplemented with (in mM) 10 TRIS–HCl (pH 8.0), 0.2 NADP+, 5 $MgCl_2$, 2 isocitrate. For aconitase activity, 25 µg of mitochondria or cytosol was supplemented with (in mM) 36 TRIS–HCl (pH 7.4), 0.8 cysteine, 0.4 $MgCl_2$, 0.2 NADP+, 30 citrate. For malate dehydrogenase activity, 20 µg of mitochondria or cytosol was supplemented with (in mM) 50 TRIS–HCl (pH 7.4), 0.2 NADP+, 5 $MgCl_2$, 5 malate.

Flow cytometry for mitochondrial superoxide emission, mitochondrial content, and apoptosis in iPSC-CM

To determine mitochondrial superoxide emission in iPSC-CM, cell cultures were stained with 5 µM MitoSOX Red (Thermo Fisher Scientific) in cell culture medium for 30 min under continued electrical field stimulation. After dissociation from the culture plates, cells were subjected to flow cytometry with excitation at 488 nm and 670 nm long pass filter for emission, calculating mean fluorescence intensity of each experimental run. For evaluation of mitochondrial superoxide emission in the absence of cardiomyocyte contraction, 2,5 µM of the myosin II ATPase inhibitor blebbistatin (Sigma-Aldrich) were added to the cell culture medium during electrical field stimulation. To determine mitochondrial content in iPSC-CM, cell cultures were stained with 100 nM MitoTracker Green FM (Thermo Fisher Scientific) for

30 min. Mean fluorescence intensity in flow cytometry was analysed with excitation at 488 nm and emission at 530 nm. To evaluate apoptosis in iPSC-CM, cells were stained with the APC Annexin V Apoptosis Detection Kit (BioLegend, San Diego, CA, USA) following the manufacturer's instructions. Cells were analysed by flow cytometry with dual excitation at 488 nm and 635 nm. Events were gated depending on APC and propidium iodide fluorescence intensity. Apoptosis rate was calculated as the percentage of events positive for APC and negative for propidium iodide. All flow cytometry experiments were run on a BD FACSCalibur and analysed using CellQuest Pro (v.5.2.1, BD Life Sciences, Franklin Lakes, NJ, USA).

Western blot

Rabbit LV tissue was homogenized in Tris buffer containing (in mM): 20 Tris-HCl, 200 NaCl, 20 NaF, 1 Na_3VO_4, 1 DTT, 1% Triton X-100 (pH 7.4), complete protease inhibitor cocktail (Roche Diagnostics, Rotkreuz, Switzerland), and phosphatase-inhibitor mixture (PhosSTOP, Roche Diagnostics). Protein concentration was determined by BCA assay (Sigma-Aldrich). Denatured proteins (30 min, 37°C) were separated on SDS-polyacrylamide gels (12.5% or 15%) and transferred to nitrocellulose membranes (GE Health Care, Chicago, IL, USA). Proteins of mitochondrial dynamics regulation were detected using anti-LC3I/II (1:1000, NB100-2331, Novus Biologicals, Littleton, CO, USA), anti-DAP1 (1:1000, NBP2-92897, Novus Biologicals), Mitochondrial Dynamics Antibody Sampler Kit II (1:2000, 74792, Cell Signaling Technology, Danvers, MA, USA, containing: anti- Tom20, anti-OPA1, anti-DRP1, anti-phospho-DRP1 (Ser616), anti-phospho-DRP1 (Ser637), anti-MFF, anti-Mitofusin-1 and anti-Mitofusin-2), and anti-GAPDH (1:10000, G8795, Sigma-Aldrich) antibodies followed by HRP-conjugated donkey anti-rabbit and sheep anti-mouse IgG antibodies (GE Health Care). To quantify levels of oxidative phosphorylation (OXPHOS) complexes, a dedicated rodent antibody cocktail targeting OXPHOS complex I-V (1:1000, ab110413, Abcam) was used. Chemiluminescent detection was performed with WesternBright Chemiluminescent Substrate (Biozym Scientific, Oldendorf, Germany). Expression levels of the proteins were normalized to GAPDH. Densitometry was performed using ImageJ (v.1.53g)

Acetylome analysis

For analysis of the mitochondrial acetylome in rabbit LV, sequential window acquisition of all theoretical mass spectra mass spectrometry (SWATH-MS) of isolated mitochondria was conducted as previously described [6]. In brief, mitochondrial isolation of LV tissue was performed by trypsin digestion, dounce homogenization, and sequential centrifugation steps with 600 to 8000 g. Precipitated proteins of mitochondrial isolates of rabbit LV tissue were reduced, carbamidomethylated and digested. 1 µg of the resulting peptide mixtures was subjected to SWATH-MS measurements. The SWATH-library was build using UniProt_TrEMBL entries for rabbit (Version 2021_01) and ProteinPilot (v.5.0, AB Sciex, Darmstadt, Germany), Paragon Method Special Factor: Acetylation emphasis. The library-search was performed as Thorough Search ID to also include missing cleavage sites. For further analysis, the SWATH-runs were then processed using PeakView (v.2.2, AB Sciex). Label free data were exported, normalized to total intensity, and filtered either for acetyl-bearing peptides or pairs of modified and unmodified peptides present in both groups SHAM and TCM.

Metabolome analysis

High performance liquid chromatography–high resolution mass spectrometry

LV tissue samples from SHAM and TCM animals were extracted using a Bead Ruptor 24 Elite homogenizer (Omni International, Kennesaw, GA, USA). Vials were filled with 960 µl methanol (HPLC-grade, prod. 34885, Honeywell, Morristown, NJ, USA), 100 µl H_2O (Milli-Q, Merck, Darmstadt, Germany) and 1.0-mm zirconia beads (cat. no. 11079110zx, BioSpec, Bartlesville, OK, USA) and pre-cooled in a water-ice bath. Frozen tissue samples and H_2O were added to a total volume of 1200 µl. Bead Ruptor cycle settings were 8.00 m/s with 30 seconds cycle time. Five cycles were used for extraction, with sample cooling times on dry ice for 2 minutes in between cycles. After the final cycle, samples were extracted at -80°C overnight. Samples were then centrifuged at 12000 g for 10 minutes at 4°C, supernatant transferred to a new protein LoBind tube (Eppendorf, Hamburg, Germany) and freeze-dried for 4.5 hours using nitrogen. Samples were reconstituted in 100 µl H_2O. 30 µl of the sample were used for measurement and 40 µl of each sample for the quality control sample pool (QC). All samples were measured in one run.

Samples and blanks (pure H_2O), QCs, and UltimateMix (UM) were measured in a stratified randomized sequence and analysed with a Dionex Ultimate 3000 HPLC (Thermo Fisher Scientific) using a reversed-phase Atlantis T3 C18 pre- and analytical column (Waters, Milford, MA, USA) with 10 µl injection volume as previously described [7]. Raw data was converted into mzXML (msConvert, ProteoWizard Toolkit v3.0.5) and detected m/z of known metabolites were identified using PeakScout (developed in-house) using a reference list containing accurate mass and retention times.

Statistical analysis was performed with R (v3.4.1, R Core Team, packages stats, FactoMineR, missMDA, nlme, lsmeans, readxl, openxlsx) and TIBCO Spotfire (v7.5.0, TIBCO, Palo Alto, USA). All metabolites were controlled for their analytical quality and graded into two classes: (I) suitable for multivariate analysis and univariate analysis (MVA_UVA) and (II) suitable for univariate analysis (UVA). Quality assessment was done as described in Vogel et al [7]. A separate control for outliers due to sequence position, weight, date of sampling, and sample extraction events was conducted via Principal Component Analysis (PCA).

To correct for weight differences and to reduce technical variability, median normalization was performed. Each metabolite was normalized to the sample median by first scaling the peak area and calculating the median. Normalized peak areas were then log-transformed. Kolmogorov-Smirnov, Brown-Forsythe Levene, Bartlett and Shapiro-Wilk tests were used to ascertain normality and homoscedasticity of data distribution. The log10-transformed, normalized data were normally distributed according to the Kolmogorov Smirnov test (98.1% of all metabolites were normally distributed) and homoscedastic according to the Brown-Forsythe Levene-type test (91.9% of all metabolites were homoscedastic).

PCA analysis was performed, centred, and scaled to unit variance (R function prcomp). Missing values were imputed by regularized expectation-maximization (R function impute PCA, estim_ncpPCA). For ANOVA analysis, the group was taken as the fixed factor (simple ANOVA; R function gls, missing values were not imputed). For specific group comparisons, pairwise post-hoc tests (R function lsmeans) were conducted. MANOVA calculation were done in RStudio (V 3.6.0), function #manova (test- Pillai; log- median- normalized data).

Technical variability was 16.6% median relative standard deviation (RSD) in the QC samples for 72 multivariate and univariate analysis (MVA_UVA) metabolites and yielded further 89 univariate analysis (UVA). The complete dataset is provided in Supplementary Datasheet 1.

Ultra-high performance liquid chromatography–high resolution mass spectrometry

For additional evaluation of metabolic alterations in early disease, metabolomic profiling was repeated including ELVD samples. LV tissue samples of SHAM, ELVD and TCM were extracted as described above. Samples and blanks (eluent B of chromatography and H_2O-ACN, 50% each), QCs, and UltimateMix (UM [7]) were measured in a stratified randomized sequence and analysed with a UHPLC Vanquish coupled to a QExactive Orbitrap system (Thermo Fisher Scientific) using an Acquity UPLC BEH Amide 1.7µm (100mm x 2,1mm; 130 Å, 186004801; Waters,) with 10 µl injection volume. Metabolite separation was achieved with a 25 min-gradient adapted from Liu et al. [8]. 90% H_2O / 10% ACN with 20mM Ammonium acetate (pH 9) was used as eluent A and 10% H_2O / 90% ACN with 20mM Ammonium acetate as eluent B. Electrospray ionization (ESI) was used for positive and negative ionization and masses between 70 and 1050 m/z were detected with a resolution of 140000 in full scan mode.

Conversion of raw data, identification of metabolites, statistical analysis, and quality assessment was done as described above; for threshold details refer to Supplemental Datasheet 2. Technical variability was 15.2% median relative standard deviation (RSD) in the QC samples for 148 multivariate and univariate analysis (MVA_UVA) metabolites and yielded further 82 univariate analysis (UVA). The log10-transformed, normalized data were normally distributed according to the Kolmogorov Smirnov test (100% of all metabolites were normally distributed) and homoscedastic according to the Brown-Forsythe Levene-type test (92.2% of all metabolites were homoscedastic). The complete dataset is provided in Supplementary Datasheet 2.

Clinical chemistry

High-sensitivity troponin T in rabbit serum samples was measured using the cobas pro platform (modules c503 and e801, Roche Diagnostics) by an immunoassay (Elecsys Troponin T hs, Roche Diagnostics). Although the assay is only certified for human sample material, we

were able to show in a preliminary study that they were also usable for rabbit samples (data not shown).

Natriuretic peptide measurements

To determine BNP gene expression in rabbit LV tissue, mRNA extraction and cDNA synthesis were performed as described above. Real-time RT-PCR was carried out in triplicate on a ViiA7 Real-Time PCR system (Thermo Fisher Scientific) using QuantiTect SYBR Green RT-PCR kit (Qiagen). GAPDH was used as housekeeping gene. To quantify cGMP tissue concentration in rabbit LV, a competitive enzyme immunoassay (cGMP ELISA kit, Cell Biolabs, San Diego, USA) was performed according to the manufacturer's instructions.

Supplementary Tables

Supplementary Table 1: Expression fold change (TCM/SHAM) of genes of mitochondrial metabolism in pathway-focused transcriptomics of rabbit left ventricular tissue.

Mitochondrial translocases are highlighted in grey.

Gene symbol	RefSeq #	Gene name	FC	p-value
AIFM2	XM_002718439	Apoptosis-inducing factor, mitochondrion-associated, 2	0.99	0.9265
AIFM3	XM_002719726	Apoptosis-inducing factor, mitochondrion-associated, 3	0.88	0.6612
AIPL1	XM_002718845	Aryl hydrocarbon receptor interacting protein-like 1	0.96	0.9487
AKT3	XM_002717648	V-akt murine thymoma viral oncogene homolog 3 (protein kinase B, gamma)	1.31	0.0774
BAK1	XM_008262789	BCL2-antagonist/killer 1-like	1.14	0.6208
BCL2L1	NM_001082135	BCL2-like 1	0.93	0.5483
BID	XM_008250128	BH3 interacting domain death agonist-like	1.26	0.7324
BNIP3	XM_002723787	BCL2/adenovirus E1B 19kD-interacting protein 3-like	0.96	0.8820
CAV2	NM_001171022	Caveolin 2	1.04	0.6846
CPT1B	XM_002723234	Carnitine palmitoyltransferase 1B (muscle)	0.78	0.1230
CPT2	XM_008265231	Carnitine palmitoyltransferase 2	0.75	0.1709
DNAJC19	XM_008266547	DnaJ homolog, subfamily C, member 19	0.98	0.7531
DNM1L	XM_002712679	Dynamin 1-like	0.94	0.5547
FIS1	XM_002722836	Mitochondrial fission 1 protein	0.75	0.0024
GCLC	XM_002714508	Glutamate-cysteine ligase, catalytic subunit	1.18	0.2575
GCLM	XM_002715852	Glutamate cysteine ligase, modifier subunit-like	1.42	0.0708
GPX1	NM_001085444	Glutathione peroxidase 1	0.85	0.2252
GRPEL2	XM_002710299	GrpE-like 1, mitochondrial-like	1.12	0.3009
HSP90AA1	XM_002721722	Heat shock 90kDa protein 1, beta	1.15	0.3729
HSPD1	XM_002712368	Heat shock 60kDa protein 1 (chaperonin)	1.08	0.5023
IMMP1L	XM_002709031	Uncharacterized LOC100358499	0.95	0.6184
IMMT	XM_008254159	Inner membrane protein, mitochondrial	0.86	0.1568
LDHA	NM_001082277	Lactate dehydrogenase A	2.12	0.0018
LOC100341303	XM_002720066	Translocase of inner mitochondrial membrane 23 (yeast) homolog	1.09	0.5155
LOC100345112	XR_085360	Translocase of inner mitochondrial membrane 17 homolog B	1.01	0.9574
LOC100345540	XM_002721675	COX16 cytochrome c oxidase assembly homolog	0.94	0.7011
LOC100348662	XM_002715995	Translocase of inner mitochondrial membrane 8 homolog A-like	1.02	0.9650
LOC100351349	XR_085313	Ras homolog gene family, member T1	0.93	0.4973
LOC100353082	XR_084882	Neurofilament, light polypeptide 68kDa	0.46	0.2935
LOC100355311	XM_002717114	Mitochondrial COX18	0.98	0.8567
LOC100355645	XM_002718611	Cytochrome oxidase assembly factor-like	0.88	0.1911
LOC100356833	XM_002718961	COX10 homolog, cytochrome c oxidase assembly protein, heme A: farnesyltransferase-like	0.88	0.1361
LOC100357146	XR_085162	Metaxin 1	0.95	0.6279
LRPPRC	XM_008254465	Leucine-rich PPR motif-containing protein	0.89	0.3021

MFN1	XM_002716333	Mitofusin 1	1.06	0.8258
MIPEP	XM_002712797	Mitochondrial intermediate peptidase	0.82	0.0811
MPV17	XM_002709955	Mpv17 protein	1.02	0.9890
MSTO1	XM_008264337	Misato homolog 1 (Drosophila)	0.95	0.5057
MTX2	XM_008258782	Metaxin 2-like	1.01	0.9632
OPA1	XM_008266674	Optic atrophy 1 (autosomal dominant)	0.93	0.3748
PMPCB	XM_002712024	Mitochondrial processing peptidase beta subunit	0.92	0.4830
PPARGC1A	XM_002709377	Peroxisome proliferator-activated receptor gamma, coactivator 1 alpha	0.86	0.3149
RNF135	XM_008271061	Ring finger protein 135-like	0.77	0.0486
SFN	XM_008265896	Stratifin-like	0.84	0.3430
SH3GLB1	XM_002715522	SH3-containing protein SH3GLB1	1.10	0.4004
SLC25A1	XM_008250237	Solute carrier family 25, member 1	0.74	0.0358
SLC25A10	XM_002724430	Solute carrier family 25 (mitochondrial carrier; dicarboxylate transporter), member 10-like	1.01	0.9388
SLC25A12	XM_008258912	Solute carrier family 25, member 12-like	0.89	0.0930
SLC25A13	XM_008261705	Solute carrier family 25, member 13-like	1.09	0.5124
SLC25A14	XM_002720292	Solute carrier family 25 (mitochondrial carrier, brain), member 14	0.95	0.5765
SLC25A15	XM_002721098	Mitochondrial ornithine transporter 1	1.10	0.4494
SLC25A16	XM_002718453	Solute carrier family 25, member 16	0.97	0.7627
SLC25A17	XM_008275178	Solute carrier family 25 (mitochondrial carrier; peroxisomal membrane protein, 34kDa), member 17	0.95	0.5082
SLC25A19	XM_002722941	Mitochondrial thiamine pyrophosphate carrier-like	0.89	0.2145
SLC25A2	XM_002710131	Solute carrier family 25 member 2-like	1.06	0.8783
SLC25A20	XM_002713398	Solute carrier family 25 (carnitine/acylcarnitine translocase), member 20	0.08	0.3055
SLC25A25	XM_008251014	Solute carrier family 25 (mitochondrial carrier; phosphate carrier), member 25	1.15	0.5245
SLC25A26	XM_008260904	Solute carrier family 25, member 26	0.98	0.7917
SLC25A27	XM_002714467	Solute carrier family 25, member 27	0.93	0.4477
SLC25A3	XM_002711214	Solute carrier family 25 member 3	0.82	0.1729
SLC25A30	XM_002712959	Solute carrier family 25, member 30	0.73	0.0683
SLC25A31	XM_002717262	Solute carrier family 25 (mitochondrial carrier; adenine nucleotide translocator), member 31	1.92	0.1086
SLC25A4	NM_001082686	Solute carrier family 25 (mitochondrial carrier; adenine nucleotide translocator), member 4	0.79	0.0412
SOD1	NM_001082627	Superoxide dismutase 1, soluble	0.78	0.0692
STARD3	XM_002719341	StAR-related lipid transfer (START) domain containing 3	0.89	0.1967
TAZ	NM_001171376	Tafazzin	0.81	0.0179
TIMM10	XM_002709160	Translocase of inner mitochondrial membrane 10 homolog	1.05	0.6057
TIMM10B	XM_002708763	Fractured callus expressed transcript 1-like	0.79	0.0830
TIMM17A	XM_002717598	Translocase of inner mitochondrial membrane 17 homolog A	1.04	0.8355
TIMM22	XM_002718896	Translocase of inner mitochondrial membrane 22 homolog	0.97	0.6517
TIMM44	XM_002722003	Translocase of inner mitochondrial membrane 44	1.00	0.9839
TIMM50	XM_008257139	Translocase of inner mitochondrial membrane 50 homolog	0.95	0.5513

TIMM8B	XM_002708437	Translocase of inner mitochondrial membrane 8 homolog b-like	1.01	0.8550
TIMM9	XM_002718273	Translocase of inner mitochondrial membrane 9 homolog	1.14	0.3808
TOMM20L	XM_002718315	Translocase of outer mitochondrial membrane 20 homolog (yeast)-like	0.97	0.7764
TOMM34	XM_002721228	Translocase of outer mitochondrial membrane 34	1.07	0.6148
TOMM40L	XM_002715147	Translocase of outer mitochondrial membrane 40-like	0.67	0.0013
TOMM5	XM_002707971	Translocase of outer mitochondrial membrane 5 homolog	0.93	0.3844
TOMM6	NM_001171253	Translocase of outer mitochondrial membrane 6 homolog (yeast)	1.06	0.6366
TOMM70A	XM_002716612	Translocase of outer mitochondrial membrane 70 homolog A (S. cerevisiae)	0.89	0.2556
TP53	NM_001082404	Tumor protein p53	1.00	0.8953
TSPO	XM_002723644	Translocator protein (18kDa)	0.82	0.7198
UCP1	NM_001171077	Uncoupling protein 1 (mitochondrial, proton carrier)	1.42	0.0877
UCP2	XM_002708700	Uncoupling protein 2	1.36	0.9216
UXT	XM_002719887	Ubiquitously-expressed transcript	0.90	0.2303

Bonferroni-adjusted α=0.00030. FC: fold change.

Supplementary Table 2: Metabolites with significant differences in multivariate analysis of variance.

Metabolite	FC ELVD	FC TCM	Pr(>F)	Pathway
3-Phosphoglyceric acid	0.55	1.40	<0.001	Gluconeogenesis
Adrenic Acid	1.59	1.18	0.005	Linolenic acid metabolism
Alanine	0.75	0.76	0.013	Glutathione metabolism, Amino acid pathways
AMP	0.60	0.69	<0.0001	Energy
Arginine	0.56	0.67	0.027	Amino acid pathways
Asparagine	0.74	0.73	0.029	Amino acid pathways
Citrate	0.84	0.91	0.040	Tricarboxylic acid cycle
Creatine	0.74	0.75	0.012	Amino acid pathways
Cytidine	0.84	1.44	0.049	Pyrimidine metabolism
Dihydroxyacetone phosphate	0.41	1.63	<0.0001	Electron transfer system, Gluconeogenesis
Docosahexaenoic acid	4.37	0.60	<0.0001	Linolenic acid metabolism
Docosapentaenoic acid	1.61	0.80	0.008	Linolenic acid metabolism
Eicosapentanoic acid	1.65	0.97	0.001	Linolenic acid metabolism
Eicosatriynoic acid	0.15	0.02	<0.001	Unsaturated fatty acids
Fumarate	0.27	0.66	0.005	Tricarboxylic acid cycle
Glutamic acid	0.78	1.10	0.027	Amino acid pathways
Glutamine	0.55	0.03	0.002	Amino acid pathways
Glycerol 3-phosphate	0.61	0.75	0.034	Electron transfer system
Guanine	0.66	1.30	0.033	Purine metabolism
Histidine	0.55	0.62	0.013	Amino acid pathways
Lysine	0.62	0.85	0.012	Amino acid pathways
Malate	0.56	0.92	<0.001	Tricarboxylic acid cycle
Methionine	0.51	0.73	0.005	Amino acid pathways
Myristoleic acid	1.94	1.17	0.003	Unsaturated fatty acids
NAD	1.58	1.26	0.012	Electron transfer system, Energy
NADH	0.11	0.64	0.000	Electron transfer system, Energy
NADPH	0.33	0.90	<0.001	Energy, Glutathione metabolism
Nonadecenoic acid	1.46	1.06	0.040	Unsaturated fatty acids
Ornithine	0.38	0.57	0.015	Amino acid pathways
Pentose	0.86	1.32	0.003	Pentose phosphate pathway
Pyroglutamic acid	0.58	0.72	0.002	Glutathione metabolism
Thymidine	16.26	7.01	0.049	Pyrimidine metabolism
Tyrosine	0.49	0.73	0.003	Amino acid pathways
Valine	1.02	0.50	0.004	Amino acid pathways
α-Ketoglutarate	0.56	0.73	<0.0001	Tricarboxylic acid cycle, Amino acid pathways

FC: fold change.

Supplementary Table 3: Fold change of metabolites of the pentose phosphate pathway and tricarboxylic acid cycle in analysis of variance.

Metabolite	ELVD/SHAM		TCM/SHAM		Pathway
	FC	Pr(>F)	FC	Pr(>F)	
Citrate	0.84	0.128	0.91	0.425	Tricarboxylic acid cycle
Fumarate	0.27	0.008	0.66	0.103	Tricarboxylic acid cycle
Malate	0.56	<0.0001	0.92	0.114	Tricarboxylic acid cycle
Succinate	1.13	0.769	0.85	0.138	Tricarboxylic acid cycle
α-Ketoglutarate	0.56	<0.001	0.33	0.032	Tricarboxylic acid cycle
6-Phosphogluconate	1.79	0.019	1.08	0.144	Pentose phosphate pathway
Gluconate	1.51	0.069	1.12	0.88	Pentose phosphate pathway
Pentose	0.86	0.128	1.32	0.30	Pentose phosphate pathway

FC: fold change.

Supplementary Figures

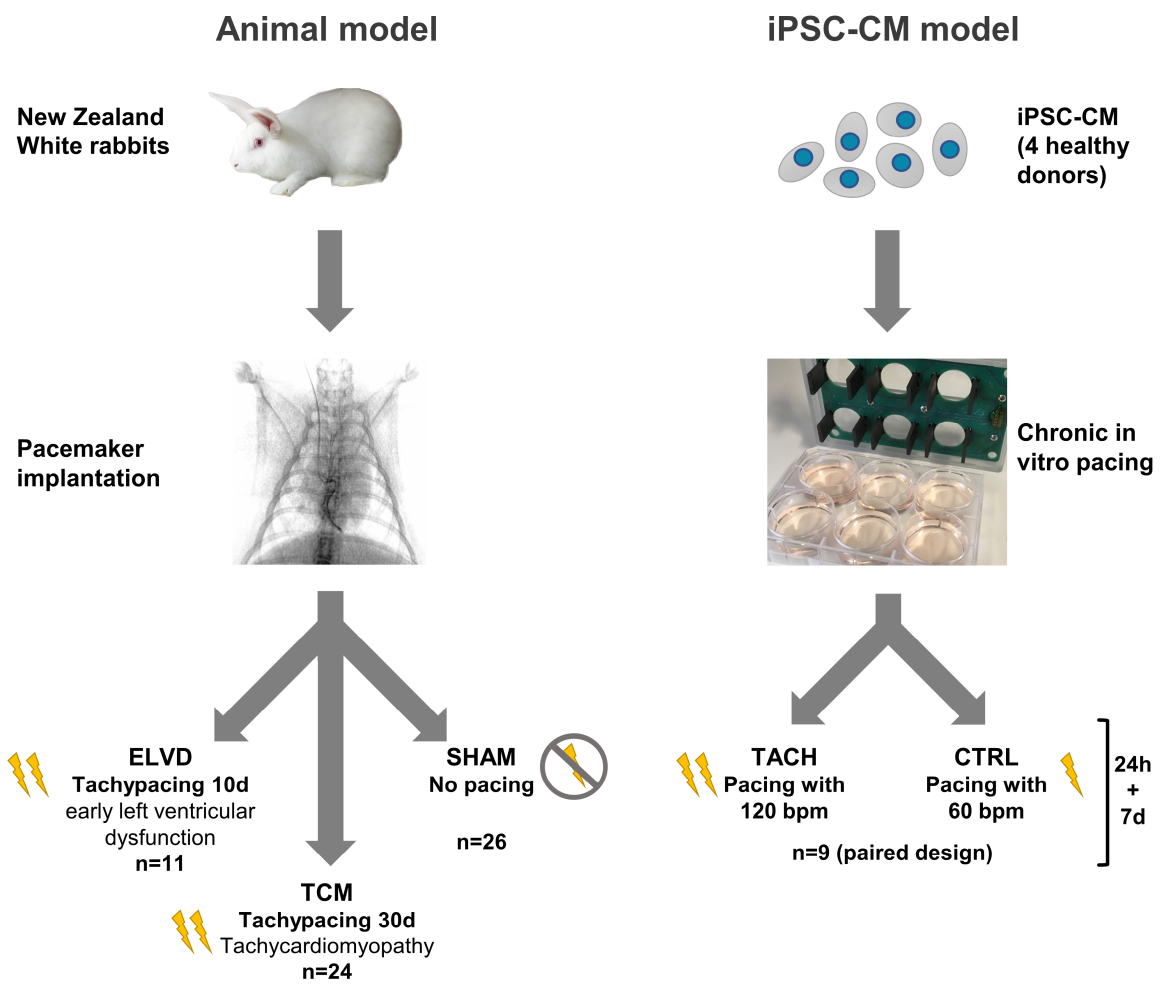

Supplementary Figure 1: Experimental study design.

iPSC-CM: human induced pluripotent stem cell derived cardiomyocytes.

With content by Otwarte Klatki, CC BY 2.0 <https://creativecommons.org/licenses/by/2.0>, via Wikimedia Commons

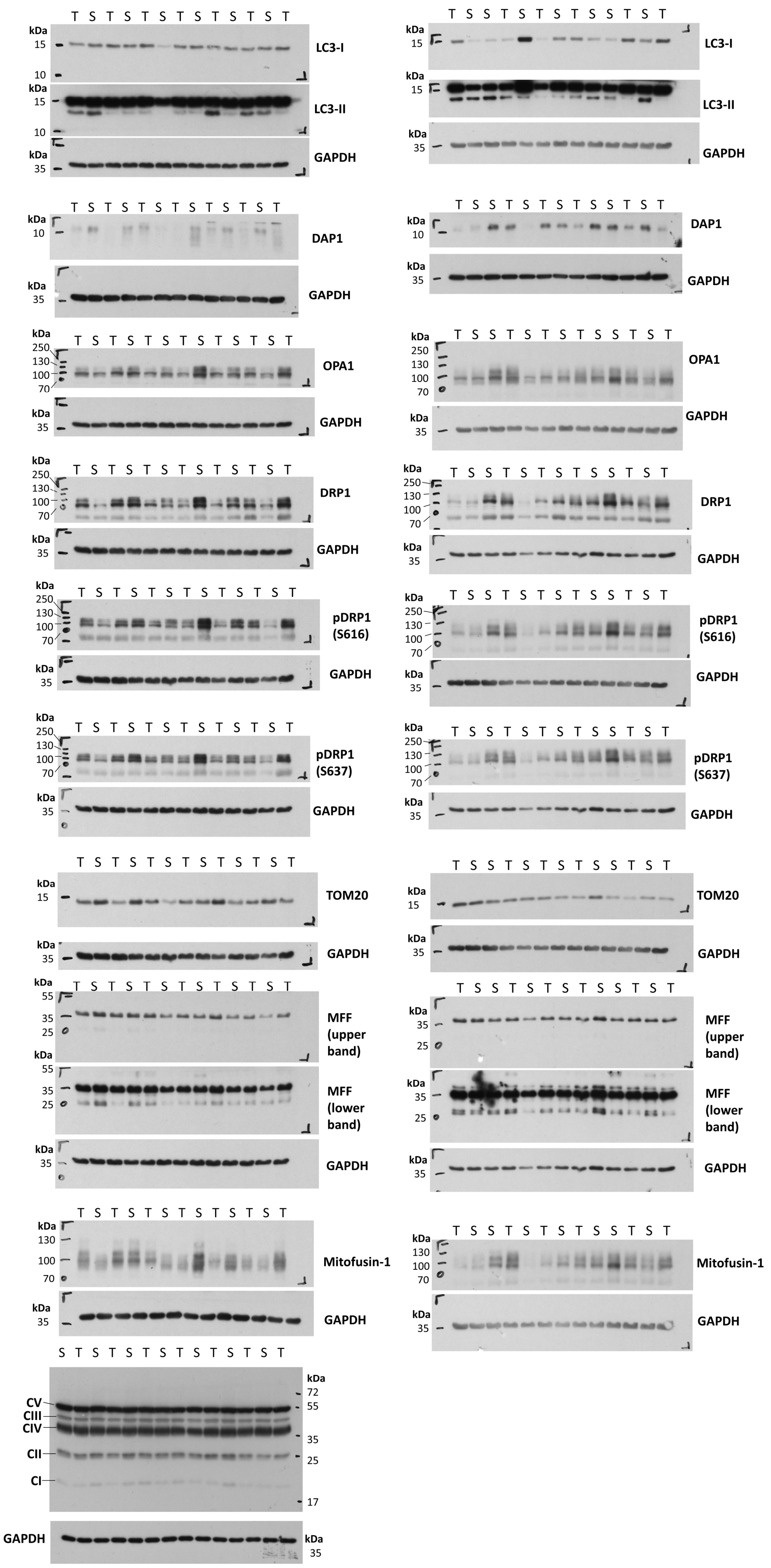

Supplementary Figure 2: Original western blot gel results. *S: SHAM. T: TCM.*

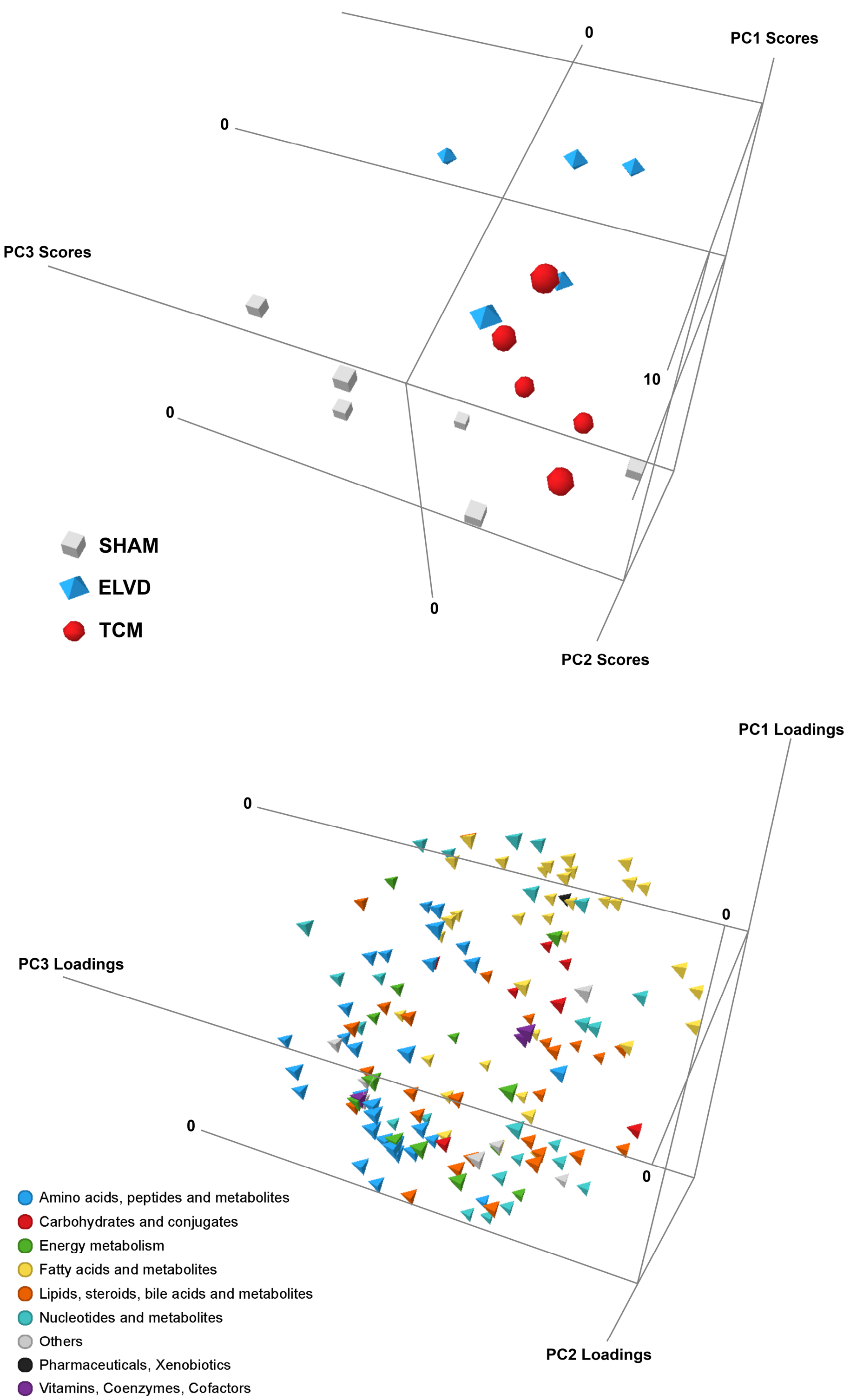

Supplementary Figure 3: Metabolomic profiling of left ventricular tissue.

Principal component analysis with scores plot (top) and loadings plot (bottom), clustering SHAM, ELVD, and TCM in three distinct groups.

PCA1 24.5%, PCA2 13.2%, PCA3 11.4%. Metabolites are coloured according to the Human Metabolome Database (HMDB) classes.

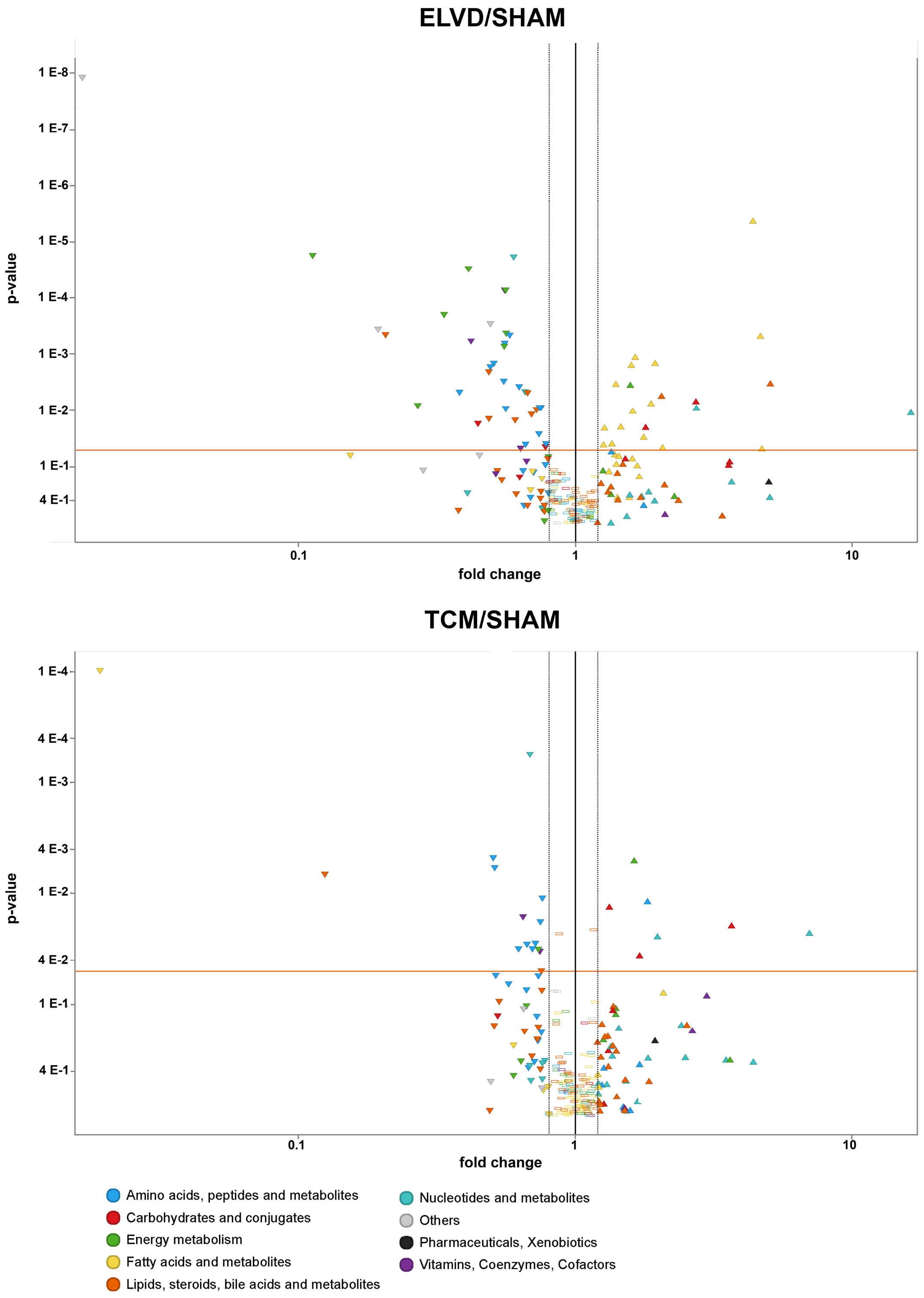

Supplementary Figure 4: Identified metabolites in left ventricular tissue. Analysis of variance volcano plot of metabolites with fold change ELVD/SHAM (top) **and TCM/SHAM** (bottom).

Y-axis: p-values (inverse log scale); x-axis: metabolite fold change (inverse log scale). Left black dotted vertical line – fold change 0.8; right black dotted vertical line – fold change of 1.2. The orange horizontal line denotes a p-value of 0.05. Metabolites are coloured according to the Human Metabolome Database (HMDB) classes.

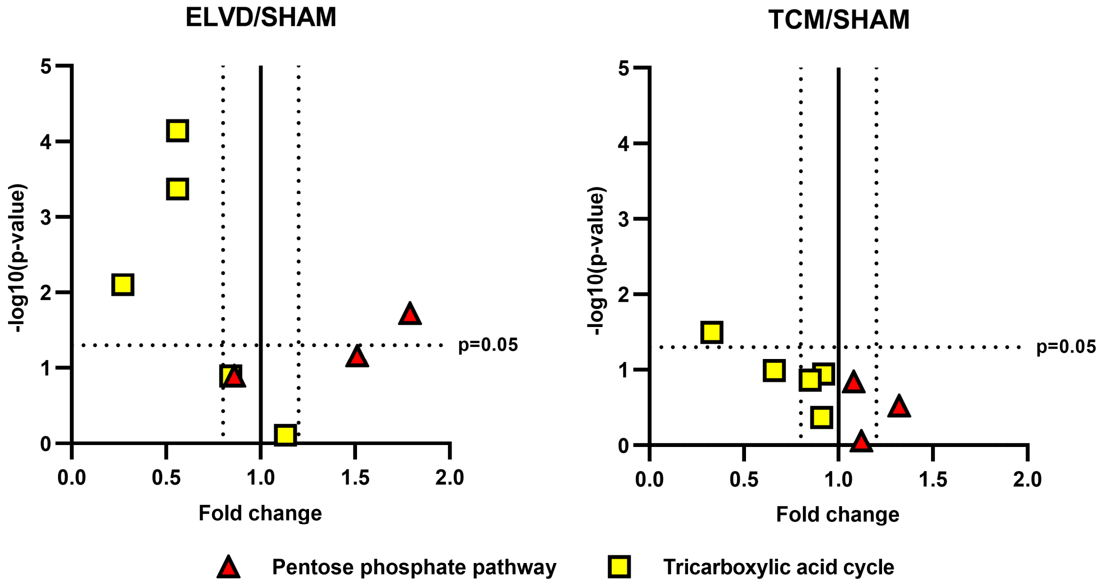

Supplementary Figure 5: Metabolic alterations in early and late stage tachycardiomyopathy encompass a depletion of tricarboxylic acid cycle metabolites, while intermediates of the pentose phosphate pathway accumulate.

Analysis of variance volcano plot of metabolites with fold change ELVD/SHAM (left) and TCM/SHAM (right) on x-axis and p-value on y-axis.

A complete list of included metabolites is provided in Supplementary Table 3.

References

1. Dietl A, Winkel I, Pietrzyk G et al. (2019) Skeletal muscle alterations in tachycardia-induced heart failure are linked to deficient natriuretic peptide signalling and are attenuated by RAS-/NEP-inhibition. PLoS One 14:e0225937. https://doi.org/10.1371/journal.pone.0225937
2. Borchert T, Hübscher D, Guessoum CI et al. (2017) Catecholamine-Dependent β-Adrenergic Signaling in a Pluripotent Stem Cell Model of Takotsubo Cardiomyopathy. J Am Coll Cardiol 70:975–991. https://doi.org/10.1016/j.jacc.2017.06.061
3. Nickel AG, Hardenberg A von, Hohl M et al. (2015) Reversal of Mitochondrial Transhydrogenase Causes Oxidative Stress in Heart Failure. Cell Metab 22:472–484. https://doi.org/10.1016/j.cmet.2015.07.008
4. Tian R, Colucci WS, Arany Z et al. (2019) Unlocking the Secrets of Mitochondria in the Cardiovascular System: Path to a Cure in Heart Failure—A Report from the 2018 National Heart, Lung, and Blood Institute Workshop. Circulation 140:1205–1216. https://doi.org/10.1161/circulationaha.119.040551
5. Krumschnabel G, Fontana-Ayoub M, Sumbalova Z et al. (2015) Simultaneous high-resolution measurement of mitochondrial respiration and hydrogen peroxide production. Methods Mol Biol 1264:245–261. https://doi.org/10.1007/978-1-4939-2257-4_22
6. Grois L, Hupf J, Reinders J et al. (2017) Combined Inhibition of the Renin-Angiotensin System and Neprilysin Positively Influences Complex Mitochondrial Adaptations in Progressive Experimental Heart Failure. PLoS One 12:e0169743. https://doi.org/10.1371/journal.pone.0169743
7. Vogel FCE, Bordag N, Zügner E et al. (2019) Targeting the H3K4 Demethylase KDM5B Reprograms the Metabolome and Phenotype of Melanoma Cells. J Invest Dermatol 139:2506-2516.e10. https://doi.org/10.1016/j.jid.2019.06.124
8. Liu Q, Cai J, Nichols RG et al. (2019) A Quantitative HILIC-MS/MS Assay of the Metabolic Response of Huh-7 Cells Exposed to 2,3,7,8-Tetrachlorodibenzo-p-Dioxin. Metabolites 9:118. https://doi.org/10.3390/metabo9060118
